# Time experience during social distancing: A longitudinal study during the first months of COVID-19 pandemic in Brazil

**DOI:** 10.1126/sciadv.abj7205

**Published:** 2022-04-13

**Authors:** André Mascioli Cravo, Gustavo Brito de Azevedo, Cristiano Moraes Bilacchi Azarias, Louise Catheryne Barne, Fernanda Dantas Bueno, Raphael Y. de Camargo, Vanessa Carneiro Morita, Esaú Ventura Pupo Sirius, Renan Schiavolin Recio, Mateus Silvestrin, Raymundo Machado de Azevedo Neto

**Affiliations:** 1 Universidade Federal do ABC (UFABC), Centro de Matemática, Computação e Cognição, Santo André, Brazil.; 2 Département Traitement de l’Information et Systèmes, ONERA, Salon-de-Provence, France.; 3 Institut de Neurosciences de la Timone, UMR7289, CNRS, Aix-Marseille Université, F13005 Marseille, France.; 4 NeurUX, Soluções em Neurociência e Tecnologia Ltda, São Paulo, Brazil.; 5 Instituto do Cérebro, IIEP-HIAE, São Paulo, Brazil.

## Abstract

Social distancing in response to the COVID-19 pandemic brought several modifications in our daily lives. With these changes, some people have reported alterations in their feelings of how fast time was passing. In this study, we assessed whether and how social distancing and the evolution of the COVID-19 pandemic influenced participants’ time awareness and production of time intervals. Participants (*n* = 3855) filled in the first questionnaire approximately 60 days after the start of social distancing in Brazil and weekly questionnaires for 15 weeks during social distancing. Our results indicate that time was perceived as expanded at the beginning, but this feeling decreased across the weeks. Time awareness was strongly associated with psychological factors such as loneliness, stress, and positive emotions, but not with time production. This relation was shown between participants and within their longitudinal reports. Together, our findings show how emotions are a crucial aspect of how time is felt.

## Teaser

This study assessed how social distancing and the evolution of the COVID-19 pandemic influenced time perception.

## INTRODUCTION

In 2020, the world faced an unprecedented health crisis with the outbreak of the severe acute respiratory syndrome coronavirus 2 pandemic. High transmission, morbidity, and mortality rates forced governments worldwide to implement social distancing measures to prevent the virus’ spread. As prolonged periods of social distancing were enforced in some parts of the world and with the evolution of the pandemic, people started reporting changes in their mental health/status, including changes in subjective time (*1*). What is commonly referred to as subjective time or temporal experience involves multidimensional and interdependent abilities such as time perspective, which is the conceptualization of a past, a present, and a future; time estimation, related to the measurement of clock time; and time awareness, which is the subjective impression of time as moving quickly or slowly (*2*–*4*).

Temporal experience is not immune to contextual and individual factors and is modulated by several factors, such as boredom, impulsivity, anxiety, routine, and emotions (*5*, *6*). As expected, it has been shown that subjective time is also affected in isolation-based experiments [e.g., people isolated in controlled environments (*7*), caves (*8*), or bunkers (*9*)]. One key aspect of these isolation-based experiments is that they are based on the radical removal of any time markers in the environment (e.g., clocks and sunlight). What resulted from coronavirus disease 2019 (COVID-19) containment measures, on the other hand, might be better defined as social distancing, requiring people to be isolated at their homes while still having access to time markers and varying degrees of social interaction. Moreover, during this period of social distancing, the pandemic continued to evolve, which led to a rise in the number of cases, increased burden on health care systems, and uncertainty of how long this situation would last. This peculiar situation allows for a more nuanced investigation of factors contributing to distortions in time estimation and time awareness.

So far, studies that investigated time perception during COVID-19 showed a mixed pattern of results. Three studies, two performed in France and one in Italy, have shown that people mainly reported the sensation that time was passing more slowly than usual, as measured with passage of time judgements tasks (*10*–*12*). On the other hand, two studies in the United Kingdom found that an equal number of participants reported that the previous day had passed more slowly or quickly, suggesting a more complex relationship between the awareness of time, social distancing, and psychological and emotional factors (*13*, *14*). The main factors associated with the sensation of time slowing down across these studies were high levels of stress, boredom and sadness, low levels of happiness, depression, young age, and dissatisfaction with social interactions (*10*–*14*). These studies, however, have three main limitations: (i) asking a single question to characterize time awareness instead of multi-item scales, (ii) no assessment of other dimensions of time experience such as time estimation, and (iii) a single assessment during the first weeks of lockdown instead of a prolonged, longitudinal assessment.

Here, we aim to characterize temporal experience during social distancing and the evolution of the COVID-19 pandemic in more detail. To assess time perception, we used both an interval production task and subjective time awareness scales. While interval productions are more directly related to time estimation abilities, time awareness scales tap into a more existential, lived experience of time. We used scales that measured two different aspects of time awareness: time expansion (with statements that refer to time feeling dilated, e.g., “I often feel bored”) and time pressure (with statements that refer to time feeling scarce, e.g., “I often think time is running out”). Although these measures can sometimes be negatively correlated, they measure different aspects of our temporal experience (*2*). For instance, a patient with a terminal disease can perceive more time pressure while still feeling a slower pace of time (*15*). These different aspects of time perception allow for a more comprehensive investigation of the subjective feelings associated with the experience of time during the COVID-19 pandemic.

Furthermore, instead of focusing only on the first weeks of social distancing, we present longitudinal data collected weekly for 5 months. In all sessions, temporal reports were combined with measures of stress, well-being, affect, and other custom-made scales, which were used to investigate which factors were more strongly associated with different aspects of temporal experience. We also collected written reports of participants’ experiences during social distancing to explore possible affective biases in the period. Combining these different measures can help the understanding of how various factors during the evolution of the pandemic are associated with time awareness.

Last, our sampled population is from Brazil, a country that suffered from severe rates of cases and deaths because of COVID-19 (as of this writing, Brazil ranks second worldwide in the number of deaths related to the pandemic). The country’s serious situation prolonged the necessity of social distancing measures and an emotional toll on its population, which might have a more detectable influence on how people perceive time. Since the government did not adopt a rigorous and homogeneous lockdown, it was possible to explore how individually embraced social distancing influenced time perception across this period.

## RESULTS

We aimed to understand whether and how social distancing and emotional and psychological factors influenced participants’ time awareness and production of time intervals during the COVID-19 pandemic. To that end, we collected data through an online survey published in social media outlets. A total of 3855 participants completed the full survey. Table S1 describes our sample’s demographic information and social distancing situation. Briefly, respondents were adult Brazilians mostly from the southeast region (80.50%), women (74.32%), highly educated (college degree, 71.78%), middle-upper class (33.08%), and working in the education (19.43%) or health care (15.36%) sectors. Although this sample is not representative of the country’s population, possibly because of the online survey format and the recruitment process, we have no reason to believe that this bias in our sample would prevent us from addressing our research questions. Most participants reported being socially isolated for 51 days when participating in the first session (median = 51, range from 0 to 120 days) at their own houses (90.56%) when they completed the first session.

To investigate whether and how time awareness changed throughout the weeks of social distancing, participants were further invited to complete weekly sessions. This questionnaire had a similar structure to the first session (more details below), without the demographic questions. Participants were informed that they did not have to fill the questionnaire every week but were asked to participate as much as possible. Weekly sessions lasted until 6 August, 14 weeks after the beginning of this study. Compared to the first session, there was a moderate dropout of participants: Of the 3855 participants in the first session, 1975 returned to complete at least one more weekly session. We had a steady decrease in the number of responses throughout the weeks (fig. S1). For the weekly sessions, we focused on data from participants that completed at least four weekly sessions (25% of the sessions), for a total of 953 participants and 7497 responses.

### Time awareness during social distancing

#### First session

Time awareness (the subjective impression of time as moving quickly or slowly) was measured using two scales (*2*): one that measured the feeling of time pressure (time feeling scarce) and the other that measured the feeling of time expansion (time pace is slow). Given that these scales were used in Brazilian Portuguese, we also investigated their construct validity with factor analysis (more details in Methods).

**Fig. 1.**
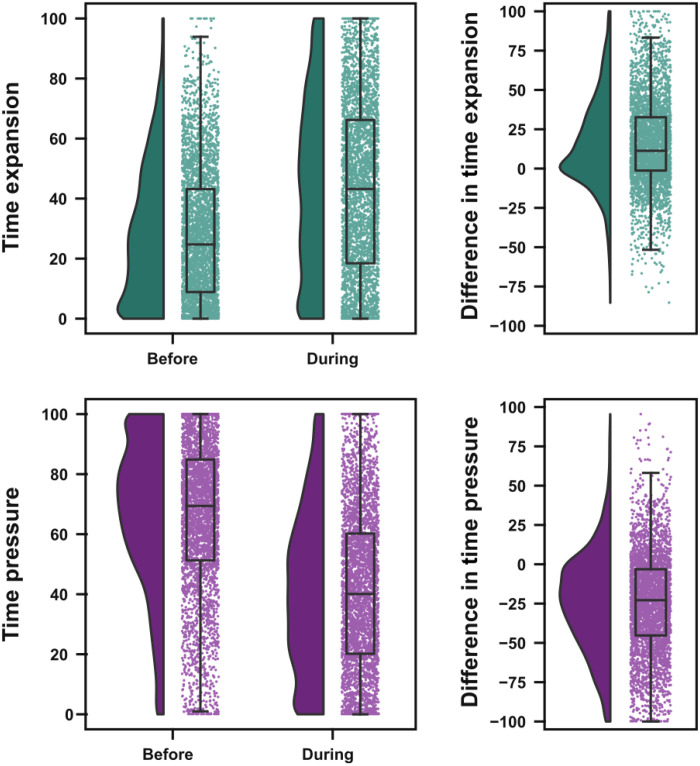
Time awareness before and during social distancing. Raincloud plots (*16*) showing group distributions as unmirrored violin plots (probability density functions), boxplots with medians and IQRs, whiskers with 5th and 95th percentiles, and individual data points (horizontally jittered). Difference plots were calculated by subtracting the reported time awareness during social distancing from the reported time awareness before social distancing.

In a first analysis, we compared time awareness during social distancing with time awareness before the pandemic [Fig. 1; raincloud plots based on (*16*)]. Participants presented strong changes in time awareness during social distancing, with an increase of temporal expansion for approximately 65% of respondents [before distancing, median (interquartile range, IQR) = 24.72 (8.85 to 43.16); during distancing, median (IQR) = 43.2 (18.46 to 66.2); both scales measured on a range from 0 to 100; Wilcoxon signed-rank test, *W* = 445051, *P* < 0.001, common language effect size (CLES) = 0.347] and a decrease of temporal pressure for approximately 75% of them [before distancing, median (IQR) = 69.40 (51.28 to 84.87); during distancing, median (IQR) = 40.08 (20.16 to 60.21); Wilcoxon signed-rank test, *W* = 903198.5, *P* < 0.001, CLES = 0.751]. There was a small, although significant, negative correlation between both time awareness measures (Shepherd rho = −0.152, *P* < 0.001).

#### Factors associated with distortions in the time awareness

To investigate possible factors that influenced time awareness, our survey included scales that measured stress and well-being [perceived stress scale (PSS-10) and World Health Organization five-item well-being index (WHO-5)] and positive and negative affect [the positive and negative affect schedule (PANAS)]. We further developed questionnaires to assess participants’ sense of loneliness, their opinions on the social distancing protocols, changes in their quality of life, and changes in their daily routine. These scales and questionnaires were combined with the demographics described in table S1 and with questions on whether participants knew someone closely related to them who had been sick because of COVID-19. A complete list of the features used with a brief description of their meanings and descriptive results can be seen in table S2.

**Fig. 2.**
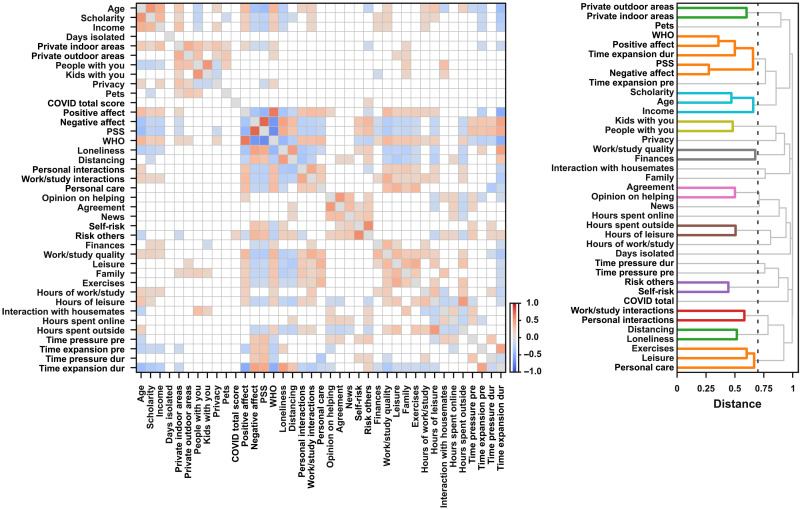
Pairwise correlations between measures and hierarchical cluster analysis in the first session. (**Left**) Pairwise Spearman correlations across measures in the first session. Only effects stronger than 0.1 are presented. The color of individual points indicates the sign and strength of the estimated coefficients. (**Right**) The estimated dendrograms using hierarchical clustering analyses of the calculated distances between scales (distance = 1 − |rho|). The black vertical lines represent the cut threshold, which was defined at a distance between scales with a correlation of medium effect size (|rho| = 0.3). Links connecting nodes with distances smaller than or equal to the threshold are marked as belonging to the same cluster.

In a first analysis, pairwise correlations between all measures were estimated across participants (Spearman’s rank correlation). To illustrate further the associations between different measures, we used hierarchical cluster analysis to build a dendrogram in which more strongly associated scales are placed on branches closer together. As can be seen in Fig. 2, there were expected associations within demographic information (e.g., age, scholarity, and income) and within information about the location in which participants were during social distancing (e.g., number of rooms in the house and number of people in the same place). Moreover, there were associations between scales used to measure emotions, stress, and well-being. Critically, time awareness seems to be more associated with scales that assess well-being, stress, and emotions.

We examined which factors were associated with stronger feelings of time expansion and pressure via a gradient boosting regression. Models were fitted on data from the first session using 5-fold cross-validation and fitted 500 times (each using a different 5-fold separation). Model performance was evaluated using three approaches: (i) Estimating the coefficient of determination (*R*^2^) between predicted and observed values. This coefficient may range up to 1 for a perfect fit and be 0 or even negative (when the mean of the data provides a better fit than the predicted values). (ii) Comparing performance with shuffled models, in which the score to be estimated (time pressure or expansion) was shuffled across participants on the training set within each fold, breaking the relationship between the variables of interest and the observed scores. (iii) Comparing performance with a baseline model, in which only time expansion and time pressure in the period before social distancing were used as predictors.

For both scales of time awareness, we found good predictive scores (measured by the coefficient of determination between predicted and observed values). For time expansion, the full model had a median *R*^2^ score of 0.53, with a minimum *R*^2^ across fits of 0.52 and a maximum *R*^2^ of 0.54. The shuffled model for time expansion, as expected, had close to chance values of *R*^2^ (median = −0.07, min = −0.12, max = −0.03), while the simpler model with only time expansion and time pressure before social distancing had lower *R*^2^ values than the complete model (median = 0.14, min = 0.12, max = 0.16). A similar pattern was found for time pressure, with good performance for the full model (median *R*^2^ = 0.25, min = 0.23, max = 0.27), chance levels for the shuffled model (median *R*^2^ = −0.08, min = −0.12, max = −0.02), and low performance for the simpler model (median *R*^2^ = 0.01, min = −0.01, max = 0.03).

Our main goal was to investigate which were the primary factors that modulated time awareness. To interpret the model’s decisions, we used Shapley additive explanations (SHAP) (*17*, *18*), which allows us to understand the role of different features in the model’s decisions. The SHAP value shows the average contribution of a feature value to the model’s prediction (*19*). Critically, SHAP values show which features the algorithm uses to make its predictions but not the actual relationship between that feature of interest and the dependent variable.

**Fig. 3.**
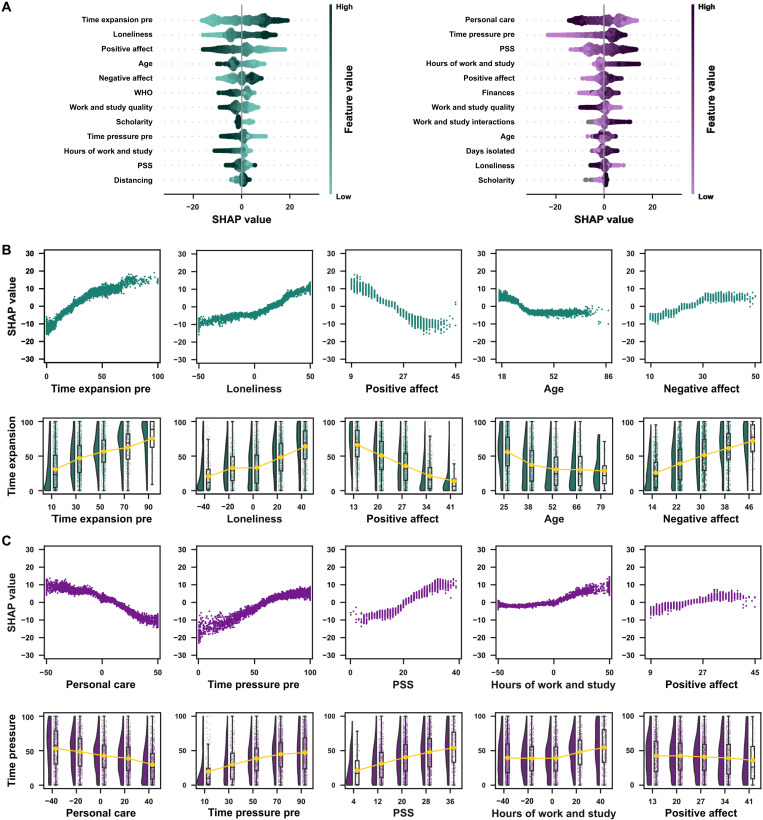
Factors associated with distortions in time awareness in the first session. (**A**) SHAP summary plots for time expansion (left) and time pressure (right). The summary plots show a combination of feature importance with feature effects. Each point is a SHAP value for a feature and an instance. Each line represents a feature, and the *x* axis is the SHAP value [on the same scale as the time awareness visual analog scale (VAS)]. The color represents the value of the feature from low to high. Overlapping points are jittered in the *y* axis direction, allowing a sense of the distribution of the SHAP values per feature. Features are sorted according to their importance. (**B**) Factors associated with distortions in time expansion. Top: SHAP dependence plots for the five features with the highest SHAP values for time expansion. Each plot shows, for each data instance, the feature value on the *x* axis and the corresponding SHAP value on the *y* axis. The SHAP value describes the behavior of the model for different values of a given feature. For example, a low score on positive affect predicted a decrease in the average value of time expansion. In contrast, high scores on positive affect predicted higher scores of time expansion. Bottom: Raincloud plots for each feature. The feature of interest is binned in five equally distanced values in each plot, and time expansion values for each bin are shown. (**C**) Factors associated with distortions in time pressure. The plots follow the same patterns as (B).

**Fig. 4.**
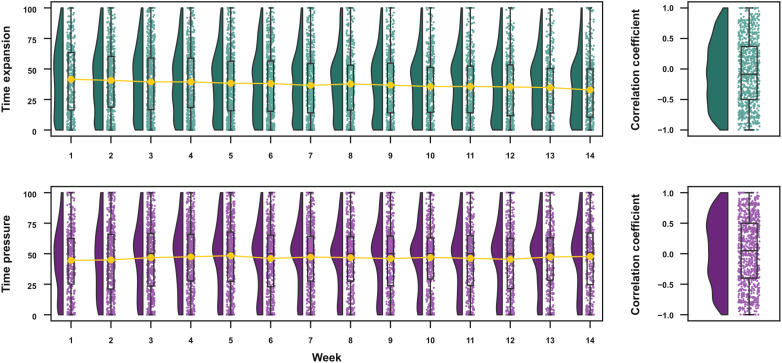
Evolution of time awareness across weeks. Raincloud plots showing group distributions as unmirrored violin plots (probability density functions), boxplots with medians and IQRs, whiskers with 5th and 95th percentiles, and individual data points (horizontally jittered). (**Right**) The distribution of correlation coefficients between week and time expansion and pressure across participants.

Figure 3A shows, for both measures of time awareness, the top 25% of the features (top 12 features of 47) that had the highest SHAP values, while panels B and C of Fig. 3 show, in more detail, the SHAP values for the top 10% (5 features of 47). To illustrate further the association between features and the dependent variables, the bottom panels of Fig. 4 (B and C) show the observed time expansion and time pressure scores as a function of the features with the highest SHAP values.

To understand Fig. 3 and the relationship between SHAP values, feature values, and the dependent variable (i.e., time expansion or time pressure), consider the following example: Take the role of loneliness in modulating the model’s output about time expansion. In the SHAP summary plot (Fig. 3A), note that higher values of loneliness (darker shades of green) are associated with higher SHAP values (*x* axis). The origin point on the *x* axis represents the model’s output if all features were unknown. As can be seen, high values of loneliness add up to 10 points to the predicted score of time expansion. The relationship between loneliness and the SHAP values for this feature can be explored further in Fig. 3B, suggesting that this relation is not entirely linear. As previously mentioned, SHAP values show how each feature contributes to the model’s predictions, not the actual relationship between a given feature and the response variable (in this example, time expansion). The bottom panels from Fig. 3B (and Fig. 3C for time pressure) show each of these features binned in five equally spaced intervals with the observed time awareness scores to illustrate the relationship between the most relevant features of the model with the actual response data. As Fig. 3B suggests, the relationship pattern between the SHAP values and actual values of loneliness closely follows the association between loneliness and time expansion, and the latter indicates that increasing levels of loneliness are associated with increased perception of time slowing down.

For time expansion, the features that had a stronger influence on the models’ predictions about reported increases/decreases were as follows: (i) time expansion before social distancing, with higher reports of temporal expansion before social distancing being associated with predictions of higher reports of temporal expansion during social distancing; (ii) feeling of loneliness, with stronger feelings of loneliness being related to predictions of higher reports of time expansion; (iii) positive affect (as measured by PANAS), with lower scores of positive emotions being associated with predictions of higher reports of time expansion; (iv) age, with younger participants related to predictions of reporting higher values of time expansion; and (v) negative affect (as measured by PANAS), with higher scores of negative emotions associated with predictions of higher reports of time expansion.

For time pressure, the features that had a stronger influence on the models’ predictions about reported increases/decreases were as follows: (i) personal care, with lower values of personal care associated with predictions of higher reports of temporal pressure; (ii) time pressure in the period before social distancing, with higher reports of time pressure before social distancing being associated with predictions of higher reports of time pressure during social distancing; (iii) perceived stress (as measured by PSS-10), with higher stress levels associated with predictions of higher reports of time pressure; (iv) hours spent working and studying, with a higher number of hours related to predictions of higher values of time pressure; and (v) positive affect (as measured by PANAS), with higher scores of positive emotions associated with predictions of higher reports of time pressure.

Overall, results for the first week of the survey show that participants’ time awareness changed after social distancing measures began. This was true both for feelings of time being empty (i.e., time expansion) and feelings of time running out (i.e., time pressure). Time awareness in the first session was best predicted by time awareness before social distancing, affect, stress, feeling of loneliness, and some everyday life activities (self-care and working hours); age was the only relevant demographic.

#### Evolution of time expansion and pressure during social distancing

As the weeks of social distancing continued, we investigated whether participants’ time awareness changed over time. As a starting point to understand these fluctuations in time awareness, we estimated participants’ median absolute deviation (MAD) over weeks for time expansion and pressure as a coarse measure of response variability. Participants’ responses varied across the weeks [time expansion: median-MAD (IQR) = 6.485 (3.179, 10.359); time pressure: median-MAD (IQR) = 6.273 (3.149, 10.275)], suggesting changes in how they perceive time across weeks.

We next investigated whether there was a monotonic increase/decrease of time pressure and expansion throughout the weeks. For each participant, we calculated the Spearman correlation coefficient (rho) between time awareness scores and week and, at the group level, compared the estimated rhos against zero using a sign test (Fig. 4). There was a small decrease of time expansion throughout the weeks [median rho (IQR) = −0.086 (−0.5, 0.369), *P* = 0.001, Cohen’s *g* = −0.054], and there was no significant association between the week and time pressure [median rho (IQR) = 0.051 (−0.4, 0.5), *P* = 0.078, Cohen’s *g* = 0.029]. Together, these results suggest that, although there were weekly variations on time awareness, these variations were not well explained by a simple increase/decrease as a function of the week.

#### Factors associated with distortions in the time awareness during weekly sessions

We performed a similar characterization of the magnitude of weekly fluctuations and possible monotonic increase/decrease for all scales that participants filled in their weekly sessions. As can be seen in table S3, the groups of questions that more consistently increase/decrease during the weeks were related to changes in routine, opinions on social distancing, and quality of life. Once again, it is important to emphasize that the MAD analysis is used to verify whether there were fluctuations in participants’ responses across the weeks, but only the Spearman correlation analysis can inform whether there was a monotonic increase/decrease over weeks.

**Fig. 5.**
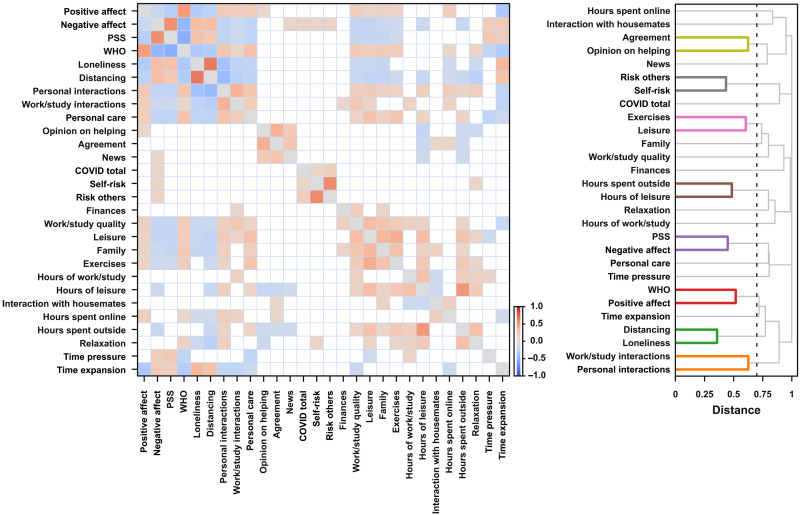
Pairwise correlations between measures and hierarchical cluster analysis during the weekly sessions. (**Left**) The median across participants of pairwise Spearman correlations for measures from weekly sessions. Only median effects stronger than 0.1 are represented. The color of individual points indicates the sign and strength of the estimated coefficients. (**Right**) The estimated dendrograms using hierarchical clustering analyses of the estimated distances between scales (distance = 1 − |rho|). The black vertical lines represent the cut threshold, which was defined at a distance between scales with a correlation of medium effect size (|rho| = 0.3). Links connecting nodes with distances smaller than or equal to the threshold are marked as belonging to the same cluster.

To investigate whether different measures covaried across weeks, we calculated, per participant, pairwise correlations of all scales that were completed weekly and estimated the median correlation across participants. To illustrate further the associations between different measures, we used hierarchical cluster analysis to build a dendrogram in which scales that are more strongly associated are placed on branches closer together (Fig. 5). Once again, weekly time expansion reports were more associated with well-being and loneliness, while time pressure was more correlated with stress and personal care.

We performed a similar gradient boosting regression to examine which factors were associated with stronger feelings of time expansion and pressure during the weekly sessions. Data from all weekly sessions were collapsed, and a single model was fit. For time expansion, once again, the full model had good predictive scores (median *R*^2^ = 0.43, min = 0.32, max = 0.52) compared to the chance levels for the shuffled model (median *R*^2^ = −0.06, min = −0.15, max = 0.03) and low performance for the simpler model (median *R*^2^ = 0.04, min = −0.17, max = 0.17). A similar pattern was found for time pressure, with good performance for the full model (median *R*^2^ = 0.27, min = 0.12, max = 0.38), chance levels for the shuffled model (median *R*^2^ = −0.06, min = −0.14, max = 0.02), and low performance for the simpler model (median *R*^2^ = −0.01, min = −0.22, max = 0.12).

**Fig. 6.**
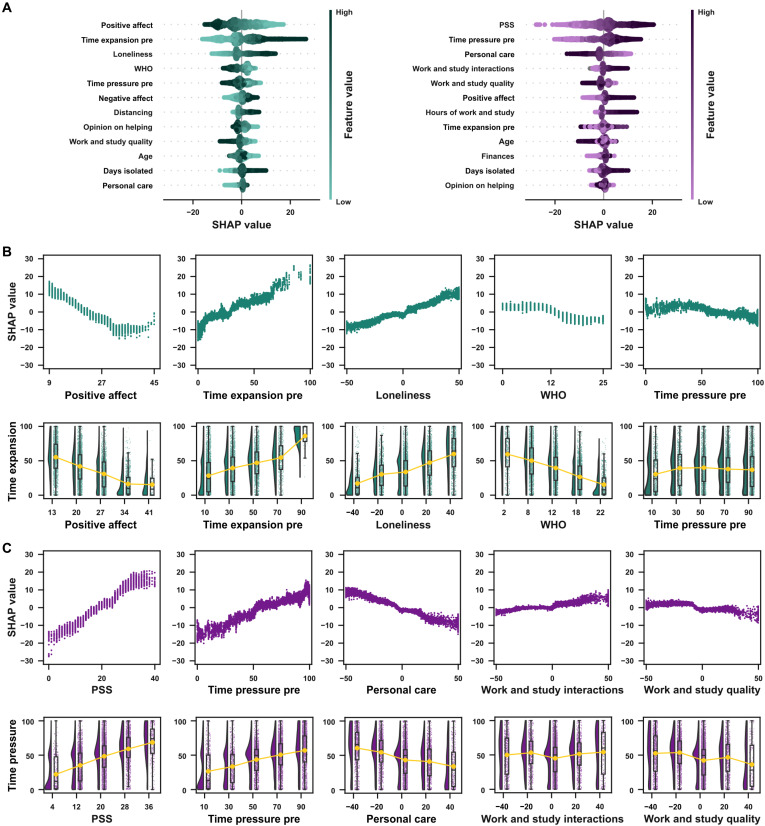
Factors associated with distortions in the time awareness during the weekly sessions. (**A**) SHAP summary plots for time expansion (left) and time pressure (right). The summary plots show a combination of feature importance with feature effects. Each point is a SHAP value for a feature and an instance. Each line represents a feature, and the *x* axis is the SHAP value (on the same scale as the time awareness VAS). The color represents the value of the feature from low to high. Overlapping points are jittered on the *y* axis direction, allowing a sense of the distribution of the SHAP values per feature. Features are ordered according to their importance. (**B**) Factors associated with distortions in time expansion. Top: SHAP dependence plots for the five features with the highest SHAP values for time expansion. Each plot shows, for each data instance, the feature value on the *x* axis and the corresponding SHAP value on the *y* axis. The SHAP value describes the behavior of the model for different values of a given feature. Bottom: Raincloud plots for each feature. The feature of interest is binned in five equally distanced values in each plot, and time expansion values for each bin are shown. (**C**) Factors associated with distortions in time pressure. The plots follow the same patterns as (B).

Figure 6 shows, for both measures of time awareness, the top 25% of the features (Fig. 6A) and the relationship in the top 10% (Fig. 6, B and C) features that had the highest SHAP values. For time expansion, the features that had a stronger influence on the models’ predictions on reported increases/decreases were as follows: (i) positive affect (as measured by PANAS), with lower scores of positive emotions being associated with predictions of higher reports of time expansion; (ii) time expansion in the period before social distancing, with higher reports of temporal expansion before social distancing being associated with predictions of higher reports of temporal expansion during social distancing; (iii) feeling of loneliness, with stronger feelings of loneliness being associated with predictions of higher reports of time expansion; (iv) well-being (as measured by WHO-5), with lower values being associated with predictions of higher reports of time expansion; and (v) time pressure in the period before social distancing, with higher reports of time pressure before social distancing being associated with predictions of lower reports of time expansion during social distancing.

For time pressure, the features that had a stronger influence on the models’ predictions on reported increases/decreases were as follows: (i) perceived stress (as measured by PSS-10), with higher stress levels being associated with predictions of higher reports of time pressure; (ii) time pressure in the period before social distancing, with higher reports of time pressure before social distancing being associated with predictions of higher reports of time pressure during social distancing; (iii) personal care, with lower values of personal care being associated with predictions of higher reports of temporal pressure; (iv) work and study interactions, with more interactions being associated with predictions of higher values of time pressure; and (v) work and study quality, with reported worse quality hours being associated with predictions of higher values of time pressure.

Overall, results from the first session and weekly sessions converge toward the same conclusion: Time awareness was distorted during social distancing. Time expansion was strongly associated with emotions and feelings of isolation, and time pressure was strongly associated with perceived stress and personal care.

#### Temporal estimates

**Fig. 7.**
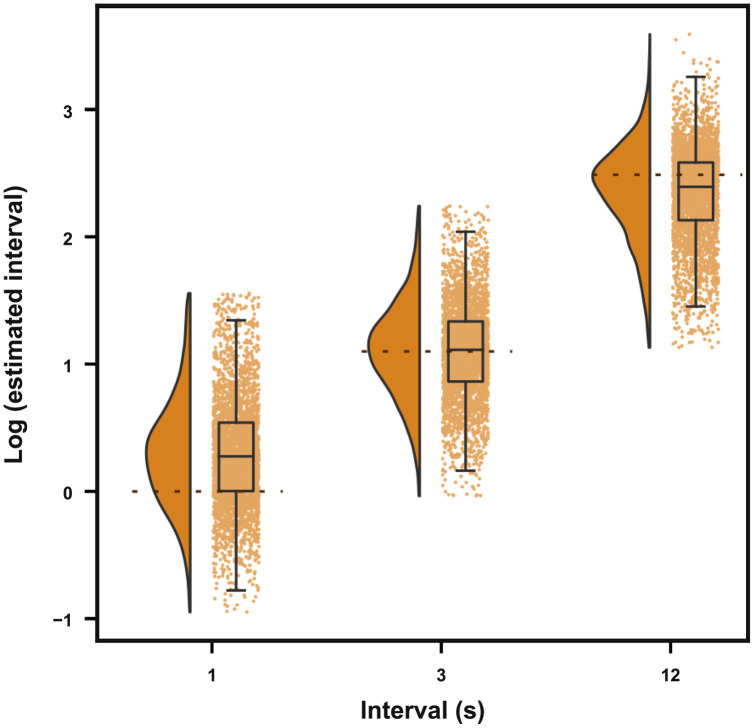
Produced intervals in the first session. Produced intervals (log-transformed) for 1, 3, and 12 s. Raincloud plots showing group distributions as unmirrored violin plots (probability density functions), boxplots with means and IQRs, whiskers with 5th and 95th percentiles, and individual data points, horizontally jittered.

Before completing the questionnaires, participants had to produce temporal intervals of 1, 3, and 12 s (three times each). Participants were prompted with a screen where they had to press a button to start the interval and press it again when they thought the corresponding time interval to be produced had ended. As shown in Fig. 7, participants were able to correctly perform the task, as evidenced by the median produced intervals being close to the target time interval and clear differences across target intervals [Friedman test: *Q*(2) = 6966.61, *P* < 0.001; paired Wilcoxon post hoc: *W*_3–12_ = 1532, *W*_3–1_ = 75449, W_12–1_ = 5; all *P* < 0.001]. Estimation accuracy differed depending on the target interval: One-second intervals were overestimated [median (IQR) = 1.32 (1 to 1.72), *P*_Holm_ < 0.001, Cohen’s *g* = 0.253], and 3-s intervals were not significantly different from 3 s [median (IQR) = 3.04 (2.37 to 3.8), *P*_Holm_ = 0.086, Cohen’s *g* = 0.014], while 12-s intervals were underestimated [median (IQR) = 10.91 (8.4 to 13.21), *P*_Holm_ < 0.001, Cohen’s *g* = −0.123].

One of our goals was to check whether time awareness was related to interval timing and whether the difference in time awareness during social distancing affected participants’ internal clocks. To measure these possible relations, we performed an across-participants Shepherd correlation (a robust version of Spearman correlation) (*20*) between the produced interval (averaged separately for 1, 3, and 12 s) and the time awareness scales. We included four scales in our analysis: (i) time pressure during social distancing, (ii) time expansion during social distancing, (iii) the difference between time pressure during and before social distancing, and (iv) the difference between time expansion during and before social distancing. These last two scales were included to investigate whether participants who had larger distortions of time awareness also had a higher distortion in temporal production. However, for all comparisons, the estimated correlations were weak and not significant [rho-range = (−0.028, 0.038); all Holm-corrected *P* > 0.08].

#### Evolution of temporal estimates across weeks

**Fig. 8.**
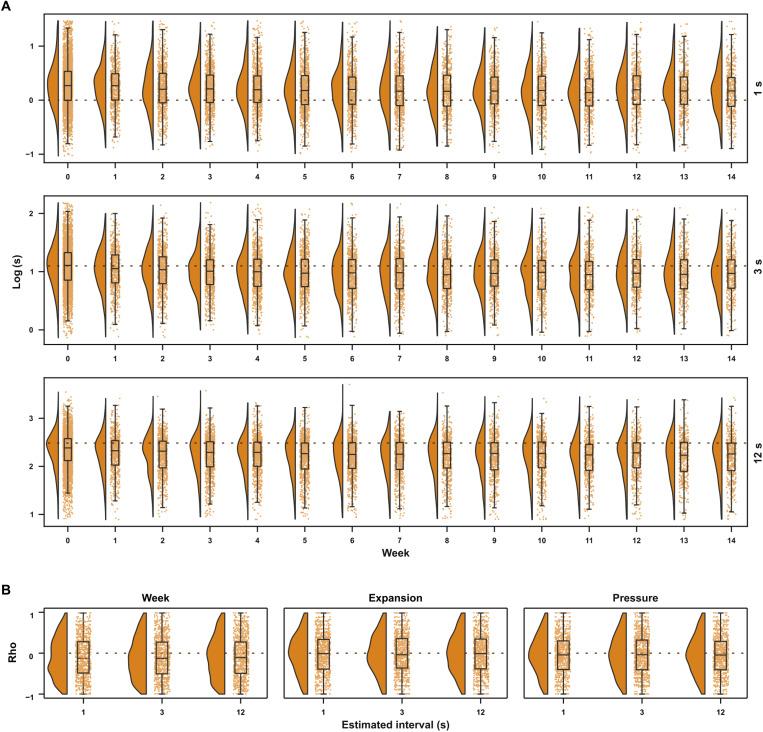
Evolution of temporal productions across weeks. (**A**) Distribution of participants’ estimates for 1-, 3-, and 12-s time intervals (log-transformed) across the weeks. (**B**) Coefficient distribution from within-participant partial Spearman correlations between temporal estimates and (i) week, considering time expansion and time pressure as covariates; (ii) time expansion with week as a covariate; and (iii) time pressure with week as a covariate.

We were interested in investigating not only whether time awareness was distorted during social distancing but also whether the production of intervals on the second’s range was affected across the weeks. Figure 8 shows the distribution of the temporal productions across the weeks. In general, participants produced shorter intervals along the weeks irrespective of the interval to be estimated [1-s rho median (IQR) = −0.12 (−0.48, 0.29), *P*_Holm_ < 0.001, Cohen’s *g* = −0.075; 3-s rho median (IQR) = −0.12 (−0.5, 0.28), *P*_Holm_ < 0.001, Cohen’s *g* = −0.092; 12-s rho median (IQR) = −0.1 (−0.49, 0.29), *P*_Holm_ < 0.001, Cohen’s *g* = −0.065].

To understand whether the decrease of the produced intervals across weeks was related to time awareness, we conducted, for each participant, Spearman partial correlations between interval productions and time awareness scores controlling for the effect of week. The produced intervals were not correlated with time awareness (Fig. 8B), neither with time expansion [1-s rho median (IQR) = 0 (−0.38, 0.35), *P*_Holm_ = 1, Cohen’s *g* = 0; 3-s rho median (IQR) = −0.03 (−0.36, 0.37), *P*_Holm_ = 0.485, Cohen’s *g* = −0.024; 12-s rho median (IQR) = 0 (−0.38, 0.36), *P*_Holm_ = 1, Cohen’s *g* = −0.008] nor with time pressure [1-s rho median (IQR) = −0.03 (−0.39, 0.31), *P*_Holm_ = 0.18, Cohen’s *g* = −0.029; 3-s rho median (IQR) = −0.03 (−0.4, 0.33), *P*_Holm_ = 0.18, Cohen’s g = −0.023; 12-s rho median (IQR) = −0.04 (−0.4, 0.3), *P*_Holm_ = 0.084, Cohen’s *g* = −0.037].

In addition, the decrease of the produced intervals across weeks was still present when controlling for the correlations with time expansion and pressure [partial Spearman correlations: 1-s rho median (IQR) = −0.1 (−0.55, 0.45), *P*_Holm_ = 0.003, Cohen’s *g* = −0.052; 3-s rho median (IQR) = −0.11 (−0.6, 0.4), *P*_Holm_ < 0.001, Cohen’s *g* = −0.063; 12-s rho median (IQR) = −0.1 (−0.6, 0.41), *P*_Holm_ = 0.004, Cohen’s *g* = −0.048] (Fig. 8B). Overall, these results indicate that the decrease of the produced intervals across weeks cannot be explained by time awareness.

### Natural language processing of open questions

#### First session

At the end of the survey, participants were invited to complete two open questions (i) to briefly share the external facts (news and events) that had marked them the most since the beginning of the social distancing and (ii) to report what had personally marked them the most since the beginning of the social distancing. We explored whether we could extract the emotional content of the responses using natural language processing (NLP) tools and whether the extracted emotional contents were correlated with time awareness. To extract the emotional context from these open-ended questions, responses were analyzed using the Stanford Sentiment Treebank (*21*) and were classified as reports with a very negative, negative, neutral, positive, or very positive polarity.

We found that the polarity of personal reports was correlated with stress (as measured by PSS-10; rho = −0.109, *P*_Holm_ < 0.001), well-being (as measured by WHO-5; rho = 0.108, *P*_Holm_ < 0.001), positive (rho = 0.103, *P*_Holm_ < 0.001) and negative emotions (rho = −0.124, *P*_Holm_ < 0.001), and time expansion during the period of social distancing (rho = −0.089, *P*_Holm_ < 0.001). The polarity of external news and facts were correlated with negative emotions (rho = −0.065, *P*_Holm_ = 0.03), stress (rho = −0.072, *P*_Holm_ = 0.01), well-being (rho = 0.086, *P*_Holm_ < 0.001), loneliness (rho = −0.077, *P*_Holm_ < 0.001), exercises (rho = 0.065, *P*_Holm_ = 0.03), and time pressure (rho = −0.075, *P*_Holm_ = 0.004) during the period of social distancing. A full report of all correlations can be seen in table S4.

#### Evolution of reports’ polarity across weeks

We investigated whether, within participants, the polarity of the reported news and personal reports were correlated with week, time awareness, and the other scales. The polarity of personal reports was correlated with positive emotions [rho median (IQR) = 0.122 (−0.222, 0.415), *P*_Holm_ < 0.001, Cohen’s *g* = 0.089] and well-being [rho median (IQR) = 0.104 (−0.17, 0.444), *P*_Holm_ < 0.001, Cohen’s *g* = 0.111]. The reported news, on the other hand, did not significantly correlate with other measures (a full report of the correlations can be seen in table S5).

Together, the NLP results showed that, in the first session, the polarity of personal reports was correlated with time expansion, while the polarity of the news and external facts was correlated with time pressure. This result agrees with the general finding of time expansion being more related to personal and psychological factors. In contrast, time pressure is more associated with a mixture of psychological and stress-related factors. We did not observe correlations between time awareness and the polarity of either personal reports or the external facts participants shared for the weekly sessions. Nevertheless, the polarity of personal reports was still correlated with general well-being measures, showing the potential power of this kind of analysis for future studies.

## DISCUSSION

This study investigated time experience during the first weeks of social distancing and of the evolution of the COVID-19 pandemic. It was the first reported longitudinal study, where data from a large group of Brazilian participants were collected during 14 weeks to understand how factors such as emotions, stress, feelings of loneliness, and daily routines modulated how participants felt the passage of time. Temporal experience was measured using time awareness scales and the production of temporal intervals. Overall, we found that the production of intervals did not change during social distancing. On the other hand, participants’ perceived time expansion increased and perceived time pressure decreased at the beginning of social distancing. As the weeks went by, the feeling of time expansion seemed to decline, while no strong modulations of time pressure were observed.

Contrary to previous studies that used passage of time judgments (*10*, *12*–*14*), we focused on time awareness scales. Specifically, the subjective impression of time before and during social distancing was estimated using scales based on previous research (*2*) that included sentences such as “I often think that time just does not want to pass” and “I haven’t enough time to complete my tasks.” Our goal, with these scales, was to quantify, in more detail, participants’ time awareness during the weeks. Specifically, when asked to judge how quickly time passes, different participants may rely on various aspects of their time awareness to make such judgments. While some may rely on the number of tasks they have to complete, others can judge how bored they are as a proxy of time slowing down. Thus, instead of using a single passage of time judgments, we have opted to measure different aspects of time awareness. As expected, on the basis of previous studies (*2*), two aspects of time awareness emerged from the items when we performed a factor analysis: time expansion (the feeling of empty time) and time pressure (the sense of time running out). Although these questions assess time perception more indirectly, they all had in common questions asking how aware participants were of their temporal experience. In general, our findings are consistent with studies that used passage of time judgments. However, future studies should investigate the relationship between these two forms of assessing time experience in more detail. One possible criticism of using these time awareness scales is that these measures and other scales that we have used, such as well-being, may ultimately refer to a common, higher-order construct, such as dimensions of psychopathology. However, an exploratory analysis did not show strong evidence in our data for an overlap between time awareness measures and well-being measures (a full description of these analyses can be seen in the Supplementary Text).

When asked about their time experience during the first weeks of social distancing, participants reported an increase in the feeling of time expansion (with approximately 65% of participants reporting this increase) and a decrease in the feeling of time pressure (with approximately 75% of participants reporting this decrease). These results express the perception of time slowing down, in line with previous studies using direct questions about the speed of passage of time (*10*–*14*). The results of the present paper and other studies investigating temporal experience during the COVID-19 pandemic indicates that, even when participants have full access to temporal markers such as clocks (*7*–*9*), sunlight, and structured daily activities, changes in time awareness occurred during the first weeks of social distancing during the COVID-19 pandemic.

The main drivers of time expansion, in our findings, were psychological factors (except age; see below), as evidenced by a strong role of emotions (both the decrease of positive emotions and the increase of negative emotions) and the feeling of loneliness. These results are in line with previous studies that found that the subjective passage of time was affected by the satisfaction of social interactions, decreased task load, increased stress and depression (*13*, *14*), increased boredom and sadness (*10*, *12*), and a decrease in happiness (*12*). Time expansion does not seem to be associated with either demographic (e.g., educational level and income) or objective data on how participants spent this period (where, with whom, and with how many people), again in line with recent studies (*10*, *12*). However, note that most of our respondents had a high income and good living conditions during the pandemic, making it impossible to generalize these findings to other, and possibly more difficult, living conditions. Participants’ age was associated with time expansion, but more so with younger participants. This effect seems to be more pronounced up to around 40 years old, after which age did not seem to play an important role. This finding is contrary to what was found in studies in the United Kingdom and France (*10*, *13*), in which older participants reported stronger slowing of time, and in the more recent French study that found no effect of age (*12*). Why age seems to have different results across studies is unclear and could be due to either cultural aspects or the usage of different scales and ways of measuring time awareness.

For time pressure, the main drivers were more equally divided between objective factors (lower scores on personal care and increased hours of work and study) and psychological factors (higher ratings of perceived stress and higher presence of positive emotions). Previous studies on time during social distancing and lockdown seem to be more related to our time expansion scores. However, Ogden (*13*) found that increased task load was associated with a feeling of a faster lockdown, consistent with our results of increased hours of work and study being associated with higher time pressure. In general, time pressure seemed to be associated with what weighed more in the balance between the cultivation of positive emotions by reserving time for personal care and both the stress of the development of new work dynamics and the danger of contamination.

What could explain these patterns of time awareness during social distancing? According to Zakay (*22*), waiting periods are judged on the basis of two central dimensions: temporal relevance (how critical it is that the thing we are waiting for occurs on time) and temporal uncertainty (how certain the timing of the event is). These two factors, in turn, will influence how much attention is given to time (temporal awareness), which will determine how slowly time will seem to pass. Increasing any of these two factors (temporal relevance or uncertainty) will lead to a slowing of time. We found that a general decrease in well-being, especially the absence of positive emotions and higher loneliness levels, was associated with the sensation of time slowing down. These findings are consistent with studies that used experience sampling and found that emotions were the main predictor of passage of time judgments (*23*, *24*).

Positive emotions have been associated with better coping mechanisms, i.e., self-regulation skills, which are essential for the psychological management of crises (*25*, *26*). Time studies in which participants had to wait with no sources of distraction for 7.5 min showed that people who were less able to emotionally self-regulate in daily life and were more impulsive felt time passing slower (*27*, *28*). It is possible that people showing lower levels of positive emotions also had trouble in dealing with the COVID-19 pandemic. The lack of internal resources could direct them to wait on external agents to ameliorate the situation, increasing the temporal relevance of the end of social distancing. Concurrently, the country’s political and sanitary situation during the sampled period was very troubled, with possible impacts on temporal uncertainty.

Although the temporal awareness approach proposed by Zakay (*22*) fits well with our findings, this model was not initially designed to explain the passage of time judgments. Most cognitive models on the perception of time distinguish two fundamental perspectives: prospective and retrospective timing. While prospective timing refers to the judgment of duration that is presently being experienced (i.e., our time production task), retrospective time perception refers to the judgment of a time interval that has already elapsed (*29*). Recently, the term “passage of time judgments” was also suggested for phenomena that do not seem to correspond with either prospective or retrospective duration judgments (*30*, *31*) but instead with the feeling of how quickly time seems to pass. Whether and how retrospective and passage of time judgments are correlated is still under debate (*30*, *31*). Future studies investigating the perceived retrospective duration of the period of social distancing can bring important advances on how these different abilities relate.

We also measured the production of time intervals in the seconds range and did not find correlations between produced intervals and time awareness. Previous studies have suggested that time awareness (as measured by scales and passage of time judgments) and temporal duration judgments (measured by timing tasks) are not directly related (*24*). On the other hand, whether levels of depression and anxiety can modulate temporal judgments is still not clear, with mixed and inconclusive findings (*5*, *32*). Our study used intervals from a wide range (of 1, 3, and 12 s) to investigate whether potential effects of psychological factors on the production of time intervals depend on the specific duration of these intervals (*5*, *32*), but we did not find consistent results for any of the tested durations. Although not necessarily unexpected, these results are important in that they suggest that time estimation models cannot account for variation on time awareness measures.

Our study was, to the best of our knowledge, the first to examine changes in participants’ responses throughout social distancing (for 14 weeks). Given that our first session took place approximately 2 months after the beginning of social distancing measures in Brazil, the results of the first session might still reflect how participants reacted to their new living conditions and to their initial impressions of time experience. Reports of time expansion had a small decrease across weeks, while no significant decrease/increase was found for time pressure. Tracking the same participants throughout the weeks of social distancing allowed us to investigate how changes in subjective and objective measures are reflected in their time awareness. In general, there was a strong consistency between the most important predictors for both time awareness scales, comparing the results of the first session with the results of the weekly sessions. The main predictors for time expansion were psychologically oriented, such as the absence of positive emotions, loneliness, and well-being. For time pressure, there was a balanced contribution between psychological factors (perceived stress and personal care) and more objective factors (how much and how satisfactory were the work/study interactions). These findings show that these factors modulate time awareness not only across participants but also within participants, with weekly fluctuations of these measures associated with concurrent modulations in time experience.

In addition to trying to understand how time experience changed during social distancing, our results also allowed us to observe how different scales evolved during this period and how these changes might influence time awareness. For example, we found a widespread improvement of well-being throughout the weeks, with decreased stress levels and negative emotions and increased well-being. Critically, during this period, Brazil’s COVID-19 cases increased and, only toward the last weeks of our research, started to stabilize. Still, participants seemed to adapt to their new conditions, as can be seen in the general increase in quality of life measures and positive changes in participants’ routines. In general, there was a tendency that seemed to go back to predistancing patterns. Participants reported spending more time outside and less time online and increasing their time dedicated to leisure. We also observed a general decrease in how much participants felt that their social distancing helped and increased the sense of being at risk. Last, we found consistent changes in their routines during the 14 weeks of social distancing. However, the improvements in daily routines were not followed by substantial concomitant changes in time awareness. This dissociation highlights, once again, how time awareness is more related to how participants are experiencing the period than to concrete changes in their routine.

One limitation of our measurement of time awareness and recent studies investigating the subjective sensation of time during the pandemic are memory recall biases (*33*). These memory biases, such as recall bias, implicit theory of change, and present state bias, are characterized by a distorted reconstruction of previous states due to their current state, knowledge, beliefs, and feelings and are common in retrospective reports (*34*). Although our participants, on average, reported an increased perception of time expansion and decreased perception of time pressure relative to the period before the pandemic, we acknowledge that their current psychological and emotional states during social distancing could have biased these reports. On the other hand, one of the advantages of our study is that this bias is likely mitigated during the longitudinal measures, given that we asked participants to assess their time awareness during that specific week.

In conclusion, we found that temporal experience changed during social distancing at the first weeks of assessment during the COVID-19 pandemic. These effects were more strongly observed in participants’ time awareness but not in their ability to produce temporal intervals. The factors more associated with time slowing down both across participants and within participants were the absence of positive emotions and loneliness. The feeling of higher time pressure was modulated by the balance between stress levels and reserving time for personal care. Together, our findings show how emotions are a crucial aspect of how time is felt.

## METHODS

### Procedure

The National Research Ethics Commission approved the experimental protocol [Certificado de Apresentação de Apreciação Ética (CAAE): 30413420.3.0000.0071], and the experiment was performed following the approved guidelines and regulations. Participants completed online questionnaires distributed through SurveyMonkey and estimated time intervals through NeurUX. The questionnaire could be completed either on a computer or cell phone. We developed two similar questionnaires, one that participants filled in their first session and a second to be completed every week. Translated and original surveys can be found at OSF. Please see the link in the “Data and materials availability” section.

### First session protocol

Participants were recruited through social media and email lists. The questionnaire was released to participants on 6 May 2020. In Brazil, social distancing protocols were uncentralized from the federal government and, thus, varied across regions in their implementation dates and restrictiveness. Most states started applying these protocols around 16 March 2020. Participants were able to join the study by performing the first session until 17 June (6 weeks after we started distributing the survey).

The first session started with participants completing a registration form at NeurUX, in which they filled their name and contact information (telephone number and email). This information was necessary so that each participant was associated with a unique ID and their responses could be combined throughout the sessions. In addition, this personal information was kept in a separate database, with only the ID number being saved into the time estimation and survey databases. Participants were then asked to estimate temporal intervals of 1, 3, and 12 s by pressing a button to start the trial and another button to stop when they thought the interval had passed. Each interval was prompted three times in random order. The interval to be estimated was displayed on the computer or smartphone screen, which also contained two buttons: a start and a stop. We provided no feedback on their performance. When the estimations were done, participants were redirected to the survey page. The software was developed in JavaScript by NeurUX enterprise, which also handled the redirection of participants to the right survey every session.

#### Measures

Participants completed the questionnaires in the order that follows.

##### Demographic questions

Participants stated their age, gender, profession, state of residence, education level, and average monthly income.

##### Social isolation situation

Participants stated the number of days in isolation, where they were staying during the pandemic, housing conditions (number of private indoor/outdoor areas), number of housemates, number of children or teenagers as housemates, the existence of a personal private room, and pet ownership.

##### COVID-19–related questions

Participants responded whether (i) they themselves, somebody in their family, or someone they knew had COVID-19–related symptoms; (ii) they themselves, somebody in their family, or someone they knew had been hospitalized because of COVID-19; and (iii) somebody in their family or someone they knew had passed away because of COVID-19.

##### Time awareness scales

Time awareness scales were based on previous work investigating subjective time perception (*2*). The scales consisted of statements on the personal experience of time to which participants stated how much they agreed on using a visual analog scale (VAS; 101 points, ranging from 0 to 100) with the anchors “not at all” and “I strongly agree.” Scales focused on two different aspects of temporal experience: time expansion and time pressure. In the first session, participants completed both scales twice, once relative to their experience of time before the social distancing period and the other relative to their experience since the beginning of social distancing. The time awareness scales consisted of the following statements. Time expansion (relative to the period before the social isolation/relative to the period during the social isolation): (i) I felt that my time seemed empty. (ii) I often felt that time just did not want to pass. (iii) I often felt bored. (iv) I often felt that I had a lot of time. (iv) I often felt that I spent my time without doing anything. Time pressure (relative to the period before the social isolation/relative to the period during the social isolation): (i) I felt that I did not have enough time to complete my tasks. (ii) I often felt time pressure. (iii) I often felt that I did have enough time to devote myself to important things. (iv) I often felt that time was running out. (v) I felt that I had to establish my priorities because I could not do everything I had to do.

##### Sense of isolation

This scale consisted of five questions in which participants reported how much the following feelings decreased or increased after their social distancing (VAS, 101 point scale, ranging from −50 to 50 with anchors “Decreased” and “Increased”): (i) loneliness, (ii) social distancing, (iii) quality of their social interactions, (iv) quality of their interactions in the work and/or study environment, and (v) self-care (personal time).

##### Opinion on the social isolation protocol

Participants stated how much they agreed on the following views about the Brazilian social isolation protocol for controlling COVID-19 (VAS, 101 point scale, ranging from −50 to 50 with anchors “Not at all” and “I strongly agree”): (i) I feel that my isolation is helping to contain the disease. (ii) I am in favor of the social isolation measures adopted so far. (iii) I have been searching and following the news about the disease. (iv) I feel that I am at risk of contamination. (v) I feel that people whom I care about are at risk of contamination.

##### Changes in the quality of life

Participants reported how much they felt that the following aspects had worsened or improved during social distancing (VAS, 101 point scale, ranging from −50 to 50 with anchors “Worsened” and “Improved”): (i) finances, (ii) work and/or study routine, (iii) leisure time, (iv) family time, and (v) exercise routine.

##### Changes in the routine related to the activity hours

Participants reported whether there was a decrease/increase during social distancing on the hours spent on the following (VAS, 101 point scale, ranging from −50 to 50 with anchors Decreased and Increased): (i) face-to-face work and/or study, (ii) face-to-face social meetings, (iii) interacting with housemates, (iv) online interactions, and (v) outdoor activities after their social isolation.

##### The positive and negative affect schedule

To evaluate affect, participants responded to a validated version of the PANAS in Brazilian Portuguese (*35*). This questionnaire provides independent indexes of positive and negative affect by considering 9-item scales (active, alert, attentive, determined, enthusiastic, excited, inspired, interested, and strong) for the positive and 10-item scales (afraid, ashamed, distressed, guilty, hostile, irritable, jittery, nervous, scared, and upset) for the negative. The “proud” item was included in the assessment but not considered in the positive index calculation because the translated word for proud is ambiguous (*35*) in Brazilian Portuguese, likely evoking a pejorative connotation. Participants responded either slightly/not at all (points = 1), a little (points = 2), moderately (points = 3), quite a bit (points = 4), or extremely (points = 5) to the items.

##### Perceived stress scale

The perception of respondents’ stress was evaluated by the PSS-10 (*36*) adapted to Brazilian Portuguese (*37*). This 10-item scale measures how much participants perceive their lives as unpredictable, uncontrollable, and overwhelming. Participants answered never (points = 0), almost never (points = 1), sometimes (points = 2), fairly often (points = 3), and very often (points = 4) to positive and negative statements. The PSS score was obtained by reversing the four positive items (items 4, 5, 7, and 8) and then summing the responses to all items (*37*).

##### World Health Organization five-item well-being index

Well-being was assessed by using the WHO-5 scale in Brazilian Portuguese (*38*). Participants answered never (points = 0), sometimes (points = 1), less than half of the time (points = 2), more than half of the time (points = 3), most of the time (points = 4), and all the time (points = 5) to the frequency of occurrence of positively worded statements at this short five-item scale.

##### Open questions

At the end of the survey, participants were invited to complete two open questions. We asked them to (i) briefly share the external facts (news and events) that have marked them most since the beginning of social distancing and (ii) report what has most marked them personally since the beginning of social distancing. Both questions were nonobligatory with an open limit of characters.

### Weekly sessions protocol

Every Thursday, participants were invited to complete a weekly session, which they had until the next Thursday to complete. These weekly sessions consisted of the temporal estimation procedure and the following questions/scales: COVID-19–related questions, time expansion, time pressure, sense of isolation, opinion on social isolation protocol, changes in the quality of life, changes in the routine related to the activity hours, PANAS, PSS-10, WHO-5, and open questions. These sessions focused on comparisons between the current and last week. This differs from the first session questionnaire, which approached the comparison between the pre- and postdistancing periods.

Starting in the seventh week (18 June), all surveys included a new question, asking about participants’ current level of social distancing, that is, what measures participants were adopting. This question was added because of the easing of restrictions on social distancing measures in Brazil at that time. In this new question, we asked the following: “Regarding your isolation level during last week.” Participants responded through a VAS with the extreme values at “my activities are home-restricted” to “I totally returned to my pre-isolation routine.”

Surveys were collected until 21 August 2020. Therefore, participants were invited to complete 15 surveys (the first session and 14 weekly sessions).

### Data analyses

All data analyses were performed using R (R Core Team, 2020; version 4.0.2) (*39*) and Python. Statistical analysis and effect sizes were performed in Python using the Pingouin library v.0.3.8 (*40*). Statistical analysis scripts are open and available at OSF.

Given that most of our variables were based on scales, most of the reported statistical analyses are based on nonparametric tests (Kruskal-Wallis, Friedman test for repeated measurements, and nonparametric correlations). When available, effect sizes are reported using the CLES (*41*, *42*) as implemented in the Pingouin library. The CLES is defined as the probability that a score sampled at random from one distribution will be greater than a score sampled from some other distribution and varies between 0 and 1.

For the temporal estimates of the first session, we corrected for multiple comparisons within each of the following family of tests: (i) post hoc comparisons of temporal estimates between the three temporal intervals, (ii) comparison of temporal estimates within each of the three temporal intervals with the reference interval, and (iii) correlation of temporal estimates with four different temporal awareness metrics (temporal expansion and pressure during social distancing and the difference between during and before the social distancing of the temporal expansion and pressure scales). For the longitudinal evaluation of temporal estimates, we corrected for multiple comparisons within each of the following family of tests: (i) correlation tests between temporal estimates across weeks for each of the three temporal intervals, (ii) partial correlations between time expansion and pressure with temporal estimates while controlling for the effect of weeks for each temporal interval, and (iii) partial correlations between temporal estimates and weeks while controlling for the effects of temporal expansion and pressure for each temporal interval.

For the longitudinal data, the *P* values of the correlations between different scales and week number were corrected on the basis of the total number of scales tested (25 scales; as can be seen in table S3). For the NLP analysis, we corrected the *P* values of the correlations between different scales and sentence polarity for the first session on the basis of the total number of scales tested (28 scales; as seen in table S4). Last, for the longitudinal analysis of sentence polarity, the *P* values of the correlations between different scales and sentence polarity were corrected on the basis of the total number of scales tested (29 scales; as seen in table S5).

For analysis investigating the correlation between variables in the first session, we used either the Spearman correlation or the Shepherd’s Pi correlation (as implemented in the Pingouin library), a robust method that returns the Spearman correlation coefficient after removing bivariate outliers (*20*, *43*). Similar results were obtained when using other robust methods, such as skipped correlation.

When investigating monotonic increases/decreases of responses as a function of week, we performed a two-stage analysis. First, we estimated the Spearman coefficients between the week and the measure of interest (e.g., time expansion, time pressure, and report polarity) per participant. Then, the estimated coefficients were compared to zero using a binomial sign test. Effect sizes for the sign test were estimated using Cohen’s *g*, as suggested in (*44*). Cohen’s *g* is used as a measure of effect size for sign tests in conditions where the expected proportion in the population is 0.5 (50%). It is calculated as the simple difference of the sample proportion in relation to the expected proportion. Positive values indicate that the observed proportion was above 0.5, and negative values indicate that it was below 0.5.

#### Preprocessing and exclusions

The first survey had 4388 registrations. We removed data from participants that did not complete the full questionnaire (*n* = 523, 12%) and participants’ IDs that were doubled (because of connection problems; *n* = 10, 0.002%). Our final sample for session 1 was 3855 participants. For the weekly surveys, we removed all data from participants that did not complete the full questionnaire and participants that did not complete session 1. All analyses on the weekly sessions were performed on data from participants that completed at least four weekly sessions (25% of the sessions) for a total of 953 participants.

#### Temporal estimates

Participants produced three temporal intervals of 1, 3, and 12 s, summing a total of nine estimates requested in random order. The selected durations were chosen to track interval timing mechanisms, given that different mechanisms are likely involved in processing intervals below and above 1 s (*45*). Three seconds is commonly related to the duration of the sense of now (*46*). Intervals of 12 s were used to look at longer time scale intervals, as used in several studies investigating time and mood disorders (*32*, *47*). Estimates lower than 0.25 s and greater than 48 s were removed, and the remaining productions were averaged according to the requested interval. Participants were excluded if all their reproductions of a single interval were considered outliers (*n* = 5). The MAD was calculated in the aggregate of participants’ log-transformed estimates separately for each interval. Participants whose average estimates were three deviations below or above the MAD in any interval were considered outliers [1-s range = (0.37, 4.81), 3-s range = (0.95, 9.07), 12-s range = (3.08, 37.91); *n* = 255] and excluded from further analysis (*48*, *49*). The final sample for temporal estimates analysis was of 3595 participants in the first session. Time estimation values were logarithmically transformed only for MAD analysis and for graphical purposes.

A similar procedure was used in the weekly sessions. Estimates lower than 0.25 s or greater than 48 s were excluded, and once more, participants were excluded if all reproductions of a single interval were considered outliers (*n* = 4). We used three deviations of MAD calculated in the aggregate of log-transformed estimates for each interval to exclude outliers, causing the indirect exclusion of participants [1-s range = (0.35, 4.35), 3-s range = (0.87, 8.89), 12-s range = (2.44, 40.98); *n* = 157]. The final sample for temporal estimate descriptive analysis was of 3694 participants in the weekly sessions. Only participants with four sessions or more were included in the regression analyses (*n* = 927). As for the first session estimations, the weekly time estimations were logarithmically transformed only for MAD analysis and graphical purposes.

#### Validated scales (PSS-10, WHO-5, and PANAS)

The scores for the PSS-10 (*37*), WHO-5 (*38*), and PANAS (*35*) were calculated according to their respective Brazilian validations. In the first session, all scales showed adequate reliability indices [McDonald’s omega (confidence interval, CI 95%): PSS-10 = 0.87 (0.63, 0.87), WHO-5 = 0.89 (0.85, 0.93), PANAS = 0.91 (0.87, 0.97); Cronbach’s alpha (CI 95%): PSS-10 = 0.87 (0.57, 0.88), WHO-5 = 0.89 (0.80, 0.92), PANAS = 0.88 (0.77, 0.95)].

#### Custom scales

Four scales were created to measure the different impacts of social distancing: sense of isolation, opinion on the social isolation protocol, changes in the quality of life, and changes in the routine related to the activity hours. These scales were developed to include items that made direct reference to the unprecedented situation of the COVID-19 pandemic. To decide whether the items of these scales should be added independently as features in our machine learning algorithm or if subsets of the items were measuring a latent construct, we used an exploratory factor analysis approach and measured the reliability of each scale. This analysis was performed on the responses of the first session, in which there were a higher number of participants. All scales showed poor factor adequacy, precluding the use of factor analysis models (KMO indices < 0.66). Furthermore, the reliability of the scales was also low (McDonald’s omega < 0.66; Cronbach’s alpha < 0.64), indicating that the scales were not unidimensional. In the face of these psychometric characteristics, raw scores were used as features.

#### Time awareness scales

Given that the time awareness scales were used in Brazilian Portuguese, we investigated their construct validity with factor analyses. We divided the first session data into even-odds partitions for exploratory and confirmatory analyses (*N*_even_ = 1928; *N*_odds_ = 1927). We expected our scale to behave similarly to the original scale in English; so in our exploratory approach, we started fitting a maximum likelihood confirmatory model with two correlated factors: time expansion and time pressure. Then, modification indexes were inspected, and modifications regarding cross-loadings for bad indicators (loadings < 0.6) were added. If the indicator still performed poorly, then it was removed from the analysis. After obtaining a robust model with the exploratory approach (see openly available code), we applied the same model to the even partition of the data in a strict confirmatory approach (i.e., without making any modifications to improve fit). These procedures were applied separately for the data from the scales with phrasings regarding the period before distancing and during distancing.

Time awareness scales in Brazilian Portuguese showed good construct validity, as assessed by factor analyses. The factors time expansion and time pressure emerged from the proposed items (all retained items loaded in one factor with loading above 0.6). Only one item in the time expansion subscale (“I often felt I had a lot of time”) was a poor indicator and was dropped. These results held for both before and during distancing item phrasings [before isolation: χ^2^(26) = 156.40, *P* < 0.001, Comparative Fit Index (CFI) = 0.99, Adjusted Goodness of Fit Index (AGFI) = 0.97, Root Mean Squared Error Approximation (RMSEA) (95% CI) = 0.05 (0.04, 0.06); during isolation: χ^2^(26) = 362.60, *P* < 0.001, CFI = 0.97, AGFI = 0.93, RMSEA (95% CI) = 0.08 (0.08, 0.09)]. Factors’ correlation was nonsignificant for the pre-isolation phrasing (rho = −0.02, *P* = 0.37). The during isolation phrasing results showed a low negative correlation (rho = −0.21, *P* < 0.001). Reliability measures were also good for both phrasings [before isolation: McDonald’s omega (95% CI) = 0.91 (0.76, 0.97), Cronbach’s alpha (95% CI) = 0.83 (0.41, 0.95); during isolation: McDonald’s omega (95% CI) = 0.92 (0.92, 0.98), Cronbach’s alpha (95% CI) = 0.86 (0.86, 0.97)].

#### Natural language processing

For both personal and external reports, we analyzed the emotional polarity content. To have a common standard, we performed a normalization that removed emojis, HTML tags, URLs, Twitter-like mentions, hashtags, enumerations, and white spaces between words. We translated all sentences to English using a Google Translate Application Programming Interface (API) for Python (https://pypi.org/project/googletrans/).

The objective of the sentiment analysis is to extract the polarity of a given sentence. For example, take the following three sentences: “They said the movie would be great, they were right.” “They said the movie would be great, they were wrong.” “There was an earthquake in Japan.” The two first sentences, although seemingly similar, have opposite polarities. The first is a positive sentence, and the second is negative, given that the movie was not great as advertised. On the other hand, the third is a neutral report of an event and does not contain positive or negative polarity.

To classify the polarity of the sentences, we used the Stanford Sentiment Treebank with an already trained recursive neural tensor network (*21*), hereafter called SST for short. The model was available using the CoreNLP Java module (https://nlp.stanford.edu/pubs/StanfordCoreNlp2014.pdf) wrapped into Stanza library (*50*) for Python. The SST receives sentences of any size and then computes an index indicating whether the sentences were very negative, negative, neutral, positive, or very positive. The main advantage of this model is to use a linguist-based heuristic, i.e., try to consider words’ position inside a sentence and their meaning. The model breaks sentences into smaller pieces and then joins them all together, creating a tree of relations. This approach is better than other standard NLP models [such as Valence Aware Dictionary and sEntiment Reasoner (VADER) or Naïve Bayes with a word vector space embedding]. These traditional models usually extract the most frequent polarity or some ratio between them, which may work on full texts but fails at the sentence level. Another advantage of the SST relative to more popular neural networks, such as Google’s Bidirectional Encoder Representations from Transformers (BERT), is the need for fewer parameters and no need for broad datasets. Models such as BERT have slow runtime and must be fine-tuned to perform the requested task and fully validated. On the other hand, the SST has lower accuracy than the modern state-of-the-art neural networks (https://paperswithcode.com/paper/recursive-deep-models-for-semantic). Together, we believe that the SST has a nice trade-off of accuracy with ease of implementation for our current proposal.

To perform the polarities’ predictions, all default values on the Stanza library were maintained. However, unlike the Java version, we cannot prevent the model from breaking line on each period, so we changed all reports’ periods to semicolons. The SST predictions consisted of five categories of polarities (very negative, negative, neutral, positive, and very positive). To illustrate what kind of sentences were classified into each category, we have chosen two sentences of each type from the weekly personal and news reports, as can be seen in Table 1.

**Table 1.** Examples of personal reports and news classified with different polarities.

	**Sentence**	**Classification**
**Personal reports**	Unfortunately, a friend’s husband died of COVID-19.	Very negative
	Surely it was my worst week, nothing good to highlight, just bad things. I do not want this feeling for anyone, especially in the middle of a pandemic.	Very negative
	The state of health of my mother-in-law.	Negative
	The pressure on teaching remotely.	Negative
	Empathy of some friendly people.	Neutral
	Think of how life will be from now on me leaves me without a prospect of improvement.	Neutral
	A great therapy session.	Positive
	I started to make crochet to sell and it was a success.	Positive
	The goals and plans I traced a few weeks ago for the pandemic seem to be working well. I slowly resumed several activities and I’m fine with my family.	Very positive
	My husband and I are well and trying to have fun a little more together, it helped me a lot.	Very positive
**News**	Unfortunately, only negative and external news are striking. The record amount of death by COVID or reopening of trade in Brazil, for example.	Very negative
	The absurd measures taken by the authorities as to the isolation and the amount of corruption not only of authorities, but in general in the acquisition, supply, and combat COVID-19.	Very negative
	Brazil epicenter of the disease.	Negative
	Social isolation loosening, even with increasing number of cases of COVID-19.	Negative
	High numbers of cases and deaths in Brazil.	Neutral
	Explosion in Beirut.	Neutral
	The hope of the vaccine increases. I hope I do not disappoint myself.	Positive
	Possible returns to face-to-face classes.	Positive
	The fact that relatives and acquaintances are well, contributes a lot to my well-being. Be able to work and help people tb.	Very positive
	The inability of the managers of the country’s various spheres to handle organized and integrated to propose and operate solutions during the pandemic; the great increase in deaths and dissemination of COVID-19 by the country.	Very positive

The examples in Table 1 were chosen to demonstrate both the results and certain limitations of the pipeline that we adopted. Because the sentences were written in Portuguese, we needed to translate them to English to use the SST. Although the automatic translation works well, some sentences do not sound natural. One could manually translate all sentences, although this could bring other types of biases.

Given that the SST is originally trained in native English and we used it in translated sentences, we aimed to validate this pipeline. To do so, we used the Brazilian Portuguese Corpus ReLi (*51*) that consists of seven book reviews, texts with one or more sentences, with manual annotation and sentence-level polarity (negative, neutral, and positive). Because the Corpus ReLi labels all words of a sentence with the same polarity, we split all reviews into sentences and excluded any sentences with words of different polarities. Then, we translated all sentences and classified them using the SST, the same strategy used with participants’ reports. Given that ReLi labels are classified only into three levels (negative, neutral, and positive), we used a similar classification for the SST, with both “very negative” and “negative” being classified as negative and “positive” and “very positive” as positive. To evaluate the classification using this pipeline, we estimated both the accuracy of the SST predictions (proportion correct = 0.45, binomial test against 0.33, *P* < 0.001) and a quadratic weighted Cohen’s κ (κ = 0.248, *P* < 0.001) (*52*), as included on the pyirr package (https://pypi.org/project/pyirr/).

For the first session reports, a Shepherd’s Pi correlation was performed between the polarities and all other scales scores. All *P* values obtained were corrected for multiple comparisons using Holm’s method. For weekly session reports, a Spearman correlation was performed between polarities and all others weekly measures for each participant. At the group level, a sign test with corrections for multiple comparisons using Holm’s method was used.

#### Exploratory data analysis and hierarchical clustering

A hierarchical clustering using a bottom-up approach was applied to the correlation between scales (distance = 1 − |rho|). This method performs a hierarchical clustering in which each scale starts in its cluster, and clusters are successively merged. The metric used for the merge strategy was the complete linkage, which minimizes the maximum distance between observations of pairs of clusters. For display purposes, distances associated with a medium effect size (|rho| ≥ 0.3) were colored using the same color.

For the weekly exploratory data analysis (Fig. 5), the pairwise correlation between weekly scales was calculated separately per participant. At the group level, the median rho for each pairwise correlation across participants was calculated. The hierarchical clustering was similar to that of session 1. A table with all correlations and uncorrected *P* values for both the first and weekly sessions can be found in the supplementary material folder in OSF.

#### Machine learning

All machine learning models used to understand the relationship between time awareness scales and other factors used the CatBoost method (*53*, *54*), an open-sourced gradient boosting library as implemented in https://github.com/catboost. CatBoost uses a gradient boosting method that can capture linear and nonlinear relations between different features and the score of interest. Given that the majority of features and dependent variables were bounded scores, linear methods are not ideal for this kind of prediction. For this reason, we opted to use an ensemble-based method. Among ensemble methods, CatBoost has performance similar to other algorithms such as LightGBM and XGBoost. However, one of the main advantages of CatBoost is that it achieves good performance using default hyperparameters, with no need to tune them. All analyses reported here used the default parameters in the CatBoostRegressor methods version 0.23.2.

Models were trained and tested using 5-fold cross-validation over the dataset. For the weekly sessions, a GroupKFold (with *K* = 5) with non-overlapping groups was used. This variant guarantees that the data of a given participant will not appear in two different folds, avoiding that the same participant is included both at the training and test sets in a given iteration.

Model performance was evaluated by estimating the coefficient of determination (*R*^2^) between predicted and observed values. This coefficient may have a maximum value of 1 for a perfect fit and a minimum value of 0 or even negative values (when the mean of the data provides a better fit than the predicted values). The *R*^2^ was estimated on the test set of each fold and averaged across folds for each model. To estimate how stable the performance was, we fit 500 iterations of the model and report the median, minimum, and maximum *R*^2^. For session 1, these 500 iterations used different fivefold separations. For the weekly sessions, a group shuffle split was used in which, in each iteration, 20% of the participants were allocated on the test set and the other 80% were allocated on the training set.

To evaluate model performance, we also calculated a permutation test score, which represents how likely an observed performance of the classifier would be obtained by chance. To generate the null distribution of performance, the *R*^2^ metric was measured in 500 different permutations of the dataset. In each permutation, the labels (the time expansion or time pressure score) of the training set were randomly shuffled, thereby removing any dependency between the features and the labels. Last, performance was also compared with a baseline model, with a smaller number of features. For session 1, the features included in the baseline model were restricted to time expansion and time pressure in the period before social distancing. For the weekly sessions, the baseline model consisted of time expansion and time pressure in the period before social distancing and week.

To interpret the model’s decisions, we used SHAP, a unified approach that connects cooperative game theory with local explanations to explain any machine learning model (*17*, *55*). The SHAP value shows the average contribution of a feature value to the model’s prediction or, in other words, given a current set of feature values, the contribution of a feature value to the difference between the actual prediction and the mean prediction (*19*). The goal of SHAP is to explain the prediction of an instance *x* by computing the contribution of each feature to the prediction using Shapley values. SHAP estimates from the CatBoost algorithm were estimated using internal functions of the CatBoost library and were always estimated on the test set. We report and show the SHAP values calculated for a single model. However, to assess how stable SHAP values were across different iterations and folds, supplemental figures in OSF show the distribution of SHAP values across 500 iterations. As shown, SHAP values and the most relevant features were highly consistent across iterations.
