# Supplementary material for: Time experience during social distancing: A longitudinal study during the first months of COVID-19 pandemic in Brazil
Source: Sci Adv. 2022 Apr 13;8(15):eabj7205. doi: 10.1126/sciadv.abj7205 (PMC9007501; doi:10.1126/sciadv.abj7205)
Supplement: Supplementary file 1 — Tables S1 to S7 Fig. S1 Supplementary Text [file sciadv.abj7205_sm.pdf]

Science Advances
AAAS

Supplementary Materials for

Time experience during social distancing: A longitudinal study during the first months of COVID-19 pandemic in Brazil

André Mascioli Cravo*, Gustavo Brito de Azevedo, Cristiano Moraes Bilacchi Azarias, Louise Catheryne Barne, Fernanda Dantas Bueno, Raphael Y. de Camargo, Vanessa Carneiro Morita, Esaú Ventura Pupo Sirius, Renan Schiavolin Recio, Mateus Silvestrin, Raymundo Machado de Azevedo Neto*

*Corresponding author. Email: andre.cravo@ufabc.edu.br (A.M.C.); raymundo.neto@einstein.br (R.M.d.A.N.)

Published 13 April 2022, *Sci. Adv.* **8**, eabj7205 (2022)
DOI: 10.1126/sciadv.abj7205

This PDF file includes:

Tables S1 to S7
Fig. S1
Supplementary Text

Participants					
	**MIN**	**Q1/4**	**Q2/4**	**Q3/4**	**MAX**
Age (years)	18	25	34	47	86
Isolation (days)	0	45	51	60	120
Education (%)	**< Level 1**	**< Level 2**	**< Level 3**	**< College**	**College**
	0.08	0.18	0.49	27.06	71.78
Income (%)	**0.5x**	**1x**	**2x**	**4x**	**>8x**
	4.41	14.4	18.18	21.82	33.08
Gender (%)	**She**	**He**	**Other**		
	74.32	25.03	0.42		
Residence in Brazil (%)	**S**	**SE**	**MW**	**NE**	**N**
	7.91	80.5	2.75	7.69	1.18

Company during social distancing					
	**MIN**	**Q1/4**	**Q2/4**	**Q3/4**	**MAX**
People (n)	0	1	2	3	25
Kids (n)	0	0	0	1	10
Pets (%)	**No**	**Yes**			
	43.06	56.94			

Housing conditions (areas) during social distancing					
	**MIN**	**Q1/4**	**Q2/4**	**Q3/4**	**MAX**
Indoor (n)	1	5	7	9	25
Outdoor (n)	0	1	2	3	10
Privacy (%)	**No**	**Yes**			
	11.11	88.89			

Table S1. Demographic information of participants. Participants' age in years, number of days in isolation, education levels, personal income based on minimum wage, gender, and residence in Brazil (S: South, SE: Southeast, MW: Midwest, NE: Northeast, N: North) are described in the upper panel. The middle panel illustrates participants' company during social distancing: number of people, number of kids, and whether they had pets. The bottom panel describes their housing condition during social distancing: number of indoor areas, number of outdoor areas, and whether they had a place to be on their own to have some privacy.

Yes-No Questions			
	**Score (Proportion of participants)**	**Theoretical Range**	**Meaning**
**COVID symptoms myself**	0.08	0-1	The proportion of participants who presented COVID symptoms themselves
**COVID symptoms family**	0.16	0-1	The proportion of participants who had someone of their family with COVID symptoms
**COVID symptoms friend**	0.18	0-1	The proportion of participants who had a friend with COVID symptoms
**COVID hospital myself**	0.00	0-1	The proportion of participants who were hospitalized due to COVID
**COVID hospital family**	0.06	0-1	The proportion of participants who had someone in their family hospitalized due to COVID
**COVID hospital friend**	0.11	0-1	The proportion of participants who had a friend hospitalized due to COVID
**COVID deaths family**	0.02	0-1	The proportion of participants who had a family member who died of COVID
**COVID deaths friend**	0.07	0-1	The proportion of participants who had a friend who died of COVID
**Scale Questions**			
	**Score (Median and IQR)**	**Theoretical Range**	**Meaning**
**Covid Total Score**	0 (0 -1)	[0 8]	Sum of all yes/no COVID responses
**Affect (PANAS Scale)**			
**Positive Affect**	23.0 (18.0-28.0)	[9 45]	Positive emotions (lower/higher values indicate lower/higher positive emotions)
**Negative Affect**	24.0 (18.0-31.0)	[10 50]	Negative emotions (lower/higher values indicate lower/higher negative emotions)
**Stress and Well-Being**			
**PSS**	21.0 (16.0-26.0)	[0 40]	Stress (lower/higher values indicate lower/higher stress levels)
**WHO**	12.0 (8.0-16.0)	[0 25]	Well being (lower/higher values indicate lower/higher well-being)
**Sense of Isolation**			
**Loneliness**	9.0 (-1.0-29.0)	[-50 +50]	How much participants' feeling of loneliness decreased/increased
**Distancing**	26.0 (11.0-46.0)	[-50 +50]	How much participants' feeling of distancing decreased/increased
**Personal Interactions**	-10.0 (-28.0-13.0)	[-50 +50]	How much participants' personal interactions decreased/increased
**Work and study interactions**	-13.0 (-30.0-8.0)	[-50 +50]	How much participants' work/study interactions decreased/increased
**Personal Care**	8.0 (-21.0-27.0)	[-50 +50]	How much participants' time for personal care (e.g., hobbies) decreased/increased
**Opinion on social distancing**			
**Opinion on helping**	86.0 (68.0-100.0)	[0 100]	How much participants believe their social distancing is helping
**Agreement**	95.0 (75.0-100.0)	[0 100]	How much participants agree with the social distancing measures
**News**	78.0 (58.0-100.0)	[0 100]	How much participants were following the news about COVID
**Self Risk**	56.0 (33.0-75.0)	[0 100]	How much participants believe they are in danger of being infected by COVID
**Others' Risk**	77.0 (61.0-99.0)	[0 100]	How much participants believe loved ones are in danger of being infected by COVID
**Quality of Life**			
**Finances**	-8.0 (-27.0-1.0)	[-50 +50]	How much participants' financial condition worsened/improved

**Work and study quality**	-16.0 (-33.0-8.0)	[-50 +50]	How much participants' work/study conditions worsened/improved
**Leisure**	-31.0 (-49.0--10.0)	[-50 +50]	How much participants' leisure worsened/improved
**Family**	-7.0 (-29.0-14.0)	[-50 +50]	How much participants' family routine worsened/improved
**Exercises**	-26.0 (-49.0-0.0)	[-50 +50]	How much participants' exercises routine worsened/improved
**Routine changes**			
**Hours of work and study**	-30.0 (-50.0-0.0)	[-50 +50]	How much participants' hours in their work/study place decreased/increased
**Hours of leisure**	-49.0 (-50.0--35.0)	[-50 +50]	How much participants' hours dedicated to social events (happy hours, restaurants) decreased/increased
**Interaction with housemates**	30.0 (1.0-50.0)	[-50 +50]	How much participants' hours spent with housemates decreased/increased
**Hours spent online**	26.0 (10.5-43.0)	[-50 +50]	How much time participants spent in online interactions decreased/increased
**Hours spent outside**	-50.0 (-50.0--37.0)	[-50 +50]	How much participants' hours out in cultural events decrease/increase

Table S2. Description and summary results of the scales from the first session. Covid related questions consisted of yes/no answers about participants' contact with Covid. (2) Validated scales were used to measure emotions (PANAS), perceived stress (PSS), and well-being (WHO-5). (3) Custom visual analog scales were used to measure participants' sense of loneliness, their opinion on social distancing, and change in their quality of life and daily routine.

Feature	MAD	IQR-MAD	Median Rho	IQR	Effect size (Cohen's g)	p-value
Affect (PANAS Scale)						
Positive Affect	2.196	[1.0, 3.0]	-0.036	[-0.463, 0.359]	-0.024	0.604
Negative Affect	2.334	[1.0, 3.0]	-0.286	[-0.6, 0.158]	-0.179	<0.001*
**Stress and Well-Being**						
PSS	2.027	[1.0, 3.0]	-0.109	[-0.527, 0.257]	-0.069	<0.001*
WHO	1.496	[1.0, 2.0]	0.062	[-0.364, 0.463]	0.048	0.032*
**Sense of Isolation**						
Loneliness	7.634	[1.0, 11.5]	-0.046	[-0.4, 0.371]	-0.027	0.539
Distancing	8.242	[2.0, 12.0]	-0.188	[-0.518, 0.258]	-0.113	<0.001*
Personal Interactions	7.43	[1.5, 11.0]	0.036	[-0.336, 0.4]	0.03	0.51
Work and study interactions	7.003	[1.0, 10.5]	0.029	[-0.4, 0.4]	0.024	0.656
Personal Care	8.336	[2.0, 12.0]	0	[-0.4, 0.359]	-0.004	0.818
**Opinion on social distancing**						
Opinion on helping	5.711	[1.0, 8.5]	-0.348	[-0.677, 0.137]	-0.197	<0.001*
Agreement	8.198	[1.0, 12.0]	-0.4	[-0.725, 0.0]	-0.25	<0.001*
News	7.396	[2.0, 11.0]	-0.4	[-0.699, 0.056]	-0.223	<0.001*
Self Risk	6.614	[2.0, 9.5]	0.167	[-0.285, 0.535]	0.106	<0.001*
Others' Risk	5.476	[1.0, 8.0]	0.047	[-0.377, 0.426]	0.029	0.539
**Quality of Life**						
Finances	4.256	[0.0, 7.0]	0.027	[-0.396, 0.429]	0.019	0.656
Work and study quality	6.197	[1.0, 9.0]	0.094	[-0.321, 0.441]	0.058	0.005*
Leisure	6.168	[1.0, 9.5]	0.11	[-0.348, 0.516]	0.066	0.001*
Family	6.23	[1.0, 10.0]	0.067	[-0.336, 0.467]	0.052	0.019*
Exercises	6.414	[0.5, 10.0]	0.082	[-0.331, 0.476]	0.045	0.056
**Routine changes**						
Hours of work and study	6.15	[0.0, 9.0]	0.082	[-0.318, 0.447]	0.058	0.007*
Hours of leisure	3.463	[0.0, 4.5]	0.3	[-0.101, 0.65]	0.192	<0.001*
Interaction with housemates	5.658	[0.0, 8.5]	-0.143	[-0.498, 0.219]	-0.098	<0.001*
Hours spent online	7.321	[2.0, 11.0]	-0.182	[-0.516, 0.224]	-0.11	<0.001*
Hours spent outside	3.829	[0.0, 4.5]	0.258	[-0.188, 0.61]	0.166	<0.001*
Relaxation	7.315	[2.75, 11.75]	0.296	[-0.289, 0.701]	0.155	<0.001*

Table S3. Evolution of the different scales during social distancing. Description of the groups of questions that more consistently increased/decreased during the weeks. The first two columns (MAD and IQR-MAD) show the variation of that measure across weeks. Median Rho and IQR shows the median Rho between that measure and week across participants. We used a binomial sign test across participants to test whether there was consistent increase/decrease of that measure over the weeks. Cohen's g shows the effect size (proportion of participants with a positive Rho - 0.5) and the respective p-value, corrected for multiple comparisons using Holm's method.

	Personal reports		News and external facts	
**Feature**	**rho**	**p-value**	**rho**	**p-value**
Time Awareness				
Time expansion during social isolation	-0.089	<0.001*	-0.058	0.072
Time pressure during social isolation	-0.027	1.000	-0.075	0.004*
Time expansion before social isolation	-0.018	1.000	-0.039	0.814
Time pressure before social isolation	-0.025	1.000	0.009	1.000
Affect (PANAS Scale)				
Positive Affect	0.103	<0.001*	0.042	0.63
Negative Affect	-0.124	<0.001*	-0.065	0.03*
**Stress and Well-Being**				
PSS	-0.109	<0.001*	-0.072	0.01*
WHO	0.108	<0.001*	0.086	<0.001*
**Sense of Isolation**				
Loneliness	-0.053	0.179	-0.077	<0.001*
Distancing	-0.055	0.140	-0.010	1.00
Personal Interactions	0.008	1.000	-0.003	1.00
Work and study interactions	0.023	1.000	0.025	1.00
Personal Care	0.014	1.000	0.038	0.81
**Opinion on social distancing**				
Opinion on helping	0.023	1.000	-0.012	1.00
Agreement	-0.004	1.000	-0.030	1.00
News	-0.007	1.000	-0.001	1.00
Self Risk	0.005	1.000	-0.047	0.37
Others' Risk	-0.024	1.000	-0.009	1.00
**Quality of Life**				
Finances	-0.002	1.000	0.012	1.00
Work and study quality	0.051	0.225	0.002	1.00
Leisure	-0.017	1.000	0.033	1.00
Family	-0.003	1.000	0.060	0.06
Exercises	0.014	1.000	0.065	0.03*
**Routine changes**				
Hours of work and study	-0.023	1.000	0.016	1.00
Hours of leisure	-0.012	1.000	0.023	1.00
Interaction with housemates	0.045	0.481	0.042	0.63
Hours spent online	0.016	1.000	0.045	0.45
Hours spent outside	0.003	1.000	0.039	0.81

Table S4. Correlation between the emotional valence of reports and measures scales. A Shepherd's correlation between the estimated valence of the reports across participants. The p-values were corrected for multiple comparisons using Holm's method.

	Personal reports				News and external facts			
Feature	Median Rho	IQR	Effect size (Cohen's g)	p-value	Median Rho	IQR	Effect size (Cohen's g)	p-value
**Week**								
**Week**	0.039	[-0.307, 0.359]	0.035	1	0.073	[-0.259, 0.36]	0.075	0.056
**Time Awareness**								
**Time expansion**	-0.01	[-0.354, 0.289]	-0.037	1	0	[-0.321, 0.289]	0	1
**Time pressure**	0	[-0.388, 0.296]	-0.015	1	0	[-0.316, 0.3]	-0.025	1
**Affect (PANAS Scale)**								
**Positive Affect**	0.133	[-0.222, 0.415]	0.089	<0.001*	0.015	[-0.287, 0.337]	0.036	1
**Negative Affect**	-0.093	[-0.435, 0.247]	-0.071	0.052	0	[-0.307, 0.293]	-0.031	1
**Stress and Well-Being**								
**PSS**	-0.081	[-0.413, 0.258]	-0.059	0.24	0	[-0.316, 0.315]	0.003	1
**WHO**	0.105	[-0.17, 0.444]	0.111	<0.001*	0.026	[-0.304, 0.333]	0.053	0.728
**Sense of Isolation**								
**Loneliness**	-0.057	[-0.342, 0.258]	-0.065	0.15	0	[-0.333, 0.288]	-0.028	1
**Distancing**	-0.024	[-0.371, 0.282]	-0.044	1	0	[-0.324, 0.316]	-0.009	1
**Personal Interactions**	0.034	[-0.258, 0.333]	0.057	0.322	0	[-0.308, 0.296]	0.024	1
**Work and study interactions**	0	[-0.303, 0.395]	0.032	1	0	[-0.292, 0.293]	0	1
**Personal Care**	0.035	[-0.278, 0.321]	0.042	1	0	[-0.307, 0.29]	0.002	1
**Opinion on social distancing**								
**Opinion on helping**	-0.027	[-0.319, 0.287]	-0.036	1	0	[-0.333, 0.335]	0.015	1
**Agreement**	0	[-0.333, 0.319]	-0.001	1	-0.038	[-0.332, 0.287]	-0.028	1
**News**	0	[-0.289, 0.339]	-0.003	1	-0.02	[-0.361, 0.299]	-0.031	1
**Self Risk**	0	[-0.316, 0.349]	0.007	1	0	[-0.287, 0.344]	0.032	1
**Others' Risk**	0	[-0.324, 0.258]	-0.022	1	0	[-0.272, 0.317]	0.014	1
**Quality of Life**								
**Finances**	0.008	[-0.304, 0.341]	0.042	1	0	[-0.344, 0.316]	-0.001	1
**Work and study quality**	0	[-0.261, 0.357]	0.028	1	0.044	[-0.283, 0.374]	0.063	0.27
**Leisure**	0.012	[-0.283, 0.394]	0.033	1	0	[-0.294, 0.322]	0.021	1
**Family**	0	[-0.333, 0.325]	0.01	1	0	[-0.289, 0.333]	0.032	1
**Exercises**	0.004	[-0.316, 0.334]	0.028	1	0.047	[-0.296, 0.359]	0.042	1
**Routine changes**								
**Hours of work and study**	-0.055	[-0.355, 0.316]	-0.052	0.572	0	[-0.316, 0.289]	0.015	1
**Hours of leisure**	0	[-0.3, 0.342]	0.03	1	0	[-0.281, 0.371]	0.014	1
**Interaction with housemates**	0	[-0.32, 0.353]	0.018	1	0	[-0.288, 0.338]	0.024	1
**Hours spent online**	0	[-0.289, 0.369]	0.026	1	0	[-0.306, 0.316]	0.014	1
**Hours spent outside**	0.038	[-0.315, 0.329]	0.053	0.572	0	[-0.325, 0.268]	0.002	1

**Relaxation**	0	[-0.483, 0.474]	0.024	1	0	[-0.5, 0.447]	0.02	1

Table S5. Correlation of the emotional content of open ended questions and different scales. A Spearman rho was calculated for each participant between the emotional valence of the personal reports and the new and external facts that they shared with the other collected scales. We used a binomial sign test across participants to test whether there was consistent increase/decrease of that measure over the weeks. Cohen's g shows the effect size (proportion of participants with a positive Rho - 0.5) and the respective p-value, corrected for multiple comparisons using Holm's method.

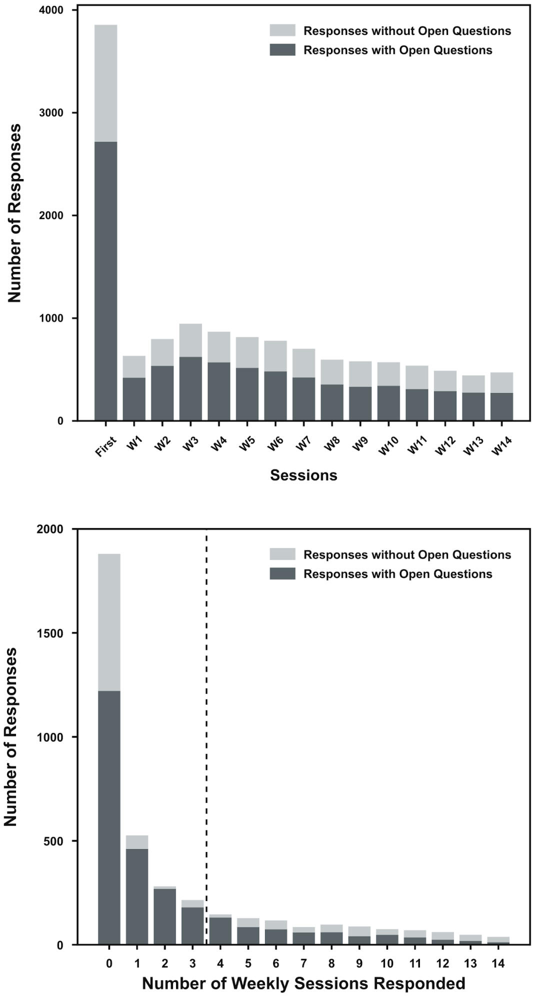

Figure S1. Number of participants and responses. Each bar on the upper panel shows the number of responses in each weekly session. On the lower panel, each bar indicates the number of respondents who completed the correspondent number of weekly sessions - zero indicates completing the first session only. The vertical dashed line indicates the inclusion criteria for weekly session analysis (participants that responded more than three times). In both panels, the dark-filled bars represent complete responses (questionnaire and open questions). In contrast, light-filled bars represent completing the survey but not the optional open questions at the end.

Supplementary Text

Exploratory analysis for an overlap between time awareness and wellbeing measures

One possible criticism of our findings is that the time awareness scales and other scales we have used, such as well-being, may ultimately refer to a common, higher-order construct, such as dimensions of psychopathology. Fortunately, our data allows a preliminary inquiry into this possibility. From a factor-analytic perspective, the hypothesis translates into a bifactor model where the time awareness items (Expansion and Pressure) and the well-being items (PSS and WHO) load both on a common ("G", or "Psychopathology") factor and on a specific factor for each instrument.

We ran three exploratory models with half of the data (as in the exploratory analyses in the manuscript), one extracting four factors (one for each scale), one with six factors (as suggested by a parallel analysis), and a bifactor model with four specific factors and one general factor. The four and six-factor models were run with the oblimin rotation and the bifactor model was run with Jenrich-Bentler bifactor rotation (also known as biquartimin). All extractions used the maximum likelihood algorithm. We show the patterns of factor loadings and the fit indexes for each model in Tables S6 and S7.

Four-Factor Solution

Item	ML1	ML4	ML2	ML3
p1	**0.86**	-0.00	-0.01	-0.04
p2	**0.84**	-0.01	0.02	0.07
p3	**0.87**	0.02	-0.01	0.05
p4	**0.93**	-0.01	0.01	-0.02
p5	**0.80**	-0.01	-0.01	0.03
e1	0.08	-0.02	0.00	**0.77**
e2	-0.06	0.01	-0.01	**0.69**
e3	0.06	0.03	-0.02	**0.79**
e4	-0.50	-0.03	-0.00	0.42
e5	-0.06	-0.02	0.03	**0.69**
eps1	-0.01	**0.77**	-0.07	-0.00
eps2	-0.01	**0.65**	0.08	-0.03
eps3	0.00	**0.78**	0.03	0.01
eps4	-0.01	-0.31	-0.38	-0.02
eps5	-0.02	-0.25	-0.34	-0.00
eps6	0.02	0.44	0.09	-0.01
eps7	-0.01	-0.44	-0.10	-0.03
eps8	-0.01	-0.35	-0.32	0.03
eps9	-0.00	**0.75**	-0.08	0.00
eps10	-0.02	**0.62**	0.10	0.01
who1	-0.01	0.27	0.56	0.02
who2	-0.00	0.38	0.48	0.01
who3	0.00	-0.03	**0.85**	0.00
who4	0.00	-0.06	**0.92**	0.00
who5	-0.01	0.01	**0.73**	-0.02

Six-Factor Solution

Item	ML2	ML3	ML4	ML1	ML5	ML6
p1	**0.86**	-0.04	0.01	-0.01	0.04	0.02
p2	**0.84**	0.07	-0.00	0.03	-0.01	-0.03
p3	**0.87**	0.04	0.00	-0.03	-0.01	0.03
p4	**0.92**	-0.02	0.01	0.03	0.00	-0.04
p5	**0.80**	0.03	-0.03	-0.05	-0.01	0.05
e1	0.08	**0.77**	-0.02	0.00	-0.01	0.00
e2	-0.06	**0.69**	-0.00	-0.02	-0.04	-0.01
e3	0.06	**0.79**	0.03	0.01	-0.02	-0.05
e4	-0.50	0.41	-0.02	-0.03	0.04	0.07
e5	-0.05	**0.68**	-0.00	0.02	0.06	0.05
eps1	-0.00	-0.01	**0.74**	0.00	0.05	0.06
eps2	-0.02	-0.02	0.48	0.04	-0.36	-0.06
eps3	0.01	0.01	**0.73**	0.01	0.03	0.18
eps4	-0.00	-0.02	0.00	-0.07	**0.63**	-0.11
eps5	-0.00	-0.01	0.07	-0.02	**0.67**	-0.07
eps6	0.01	-0.01	0.32	0.08	-0.24	-0.06
eps7	-0.00	-0.03	-0.25	0.06	0.29	-0.14
eps8	0.01	0.02	-0.05	-0.06	**0.64**	-0.02
eps9	-0.00	0.01	**0.73**	0.05	-0.00	-0.07
eps10	-0.03	0.02	0.45	0.05	-0.33	-0.04
who1	0.01	-0.01	0.07	0.16	-0.10	**0.62**
who2	0.01	-0.02	0.18	0.13	-0.14	0.53
who3	0.00	0.01	0.02	**0.83**	0.02	0.01
who4	-0.00	0.01	-0.02	**0.92**	0.00	-0.01
who5	-0.01	-0.02	0.00	**0.62**	-0.05	0.10

Bifactor-Factor Solution

Item	ML1	ML2	ML3	ML4	ML5
p1	-0.03	**0.86**	-0.06	0.00	0.03
p2	-0.00	**0.84**	0.05	0.01	-0.01
p3	0.00	**0.87**	0.02	-0.01	0.00
p4	-0.01	**0.93**	-0.04	0.00	-0.01
p5	-0.03	**0.80**	0.01	-0.01	-0.01
e1	-0.01	0.03	**0.76**	0.00	-0.01
e2	0.01	-0.10	**0.69**	-0.02	-0.02
e3	0.03	0.02	**0.79**	-0.02	-0.00
e4	-0.03	-0.53	0.43	0.02	0.03
e5	-0.00	-0.10	**0.69**	0.04	0.04
eps1	**0.64**	-0.01	-0.01	-0.06	0.40
eps2	**0.71**	-0.01	-0.03	-0.08	0.05
eps3	**0.73**	0.00	0.01	-0.00	0.41
eps4	**-0.67**	-0.01	-0.02	-0.07	0.26
eps5	**-0.60**	-0.02	-0.00	-0.04	0.31
eps6	0.51	0.02	-0.02	-0.02	0.03
eps7	-0.52	-0.00	-0.03	0.02	-0.02
eps8	**-0.67**	-0.01	0.03	-0.02	0.26
eps9	**0.62**	0.00	-0.00	-0.09	0.33
eps10	**0.69**	-0.02	0.01	-0.05	0.06
who1	**0.67**	-0.01	0.02	0.36	0.10
who2	**0.71**	-0.00	0.01	0.29	0.13
who3	**0.61**	0.00	0.00	0.58	-0.01
who4	**0.64**	-0.00	0.00	**0.62**	-0.05
who5	0.56	-0.01	-0.02	0.48	-0.03

Table S6. Factor loadings for the three models. Four-Factor solution (one for each scale); Six-Factor solution (as suggested by a parallel analysis) and the Bifactor-Factor solution. In each table, p refers to Time Pressure Items; e to Time Expansion Items; eps to PSS items; who to WHO-5 items; and ML to the factor extracted by maximum likelihood.

Fit indices

factors	chi.squared	p_value	RMSEA	TLI	BIC
4	974.47	0.00	0.06	0.93	-102.76
6	303.92	0.00	0.04	0.97	-661.43
bifactor (4+G)	394.67	0.00	0.04	0.96	-576.85

Table S7. Fit indexes for each model. Fit indexes for the Four-Factor solution (one for each scale); Six-Factor solution (as suggested by a parallel analysis) and the Bifactor-Factor solution.

All models had a good fit. The factor loadings for the four-factor solution show some items that do not strongly load on any factor. The factors that do load preferentially are always from a single scale. The same is true for the six-factor solution, although the WHO-5 and PSS items appear divided into two factors each. The bifactor solution reveals that EPS and WHO seems to both reflect a higher-order factor. However, even in this model, the time awareness items do not load onto that same latent variable and remain distinct. Overall, we see no evidence in our data for an overlap between time awareness measures and wellbeing measures, which are closer to psychopathology constructs.
